# Cationic peptides erase memories by removing synaptic AMPA receptors through endophilin-mediated endocytosis

**DOI:** 10.21203/rs.3.rs-3559525/v1

**Published:** 2023-11-21

**Authors:** Eric Stokes, Yinyin Zhuang, Michael Toledano, Jose Vasquez, Ghalia Azouz, May Hui, Isabella Tyler, Xiaoyu Shi, Jason Aoto, Kevin T. Beier

**Affiliations:** 1 Pharmacology Graduate Program, University of Colorado Anschutz, Aurora, CO 80045, USA; 2 Department of Developmental and Cell Biology, University of California, Irvine, CA 92697, USA; 3 Department of Physiology and Biophysics, University of California, Irvine, Irvine, CA, USA 92697-4560; 4 Department of Chemistry, University of California, Irvine, CA 92697, USA; 5 Department of Biomedical Engineering, University of California, Irvine, Irvine, CA, USA 92697-4560; 6 University of Colorado Anschutz, Department of Pharmacology, Aurora, CO 80045, USA; 7 Department of Neurobiology and Behavior, University of California, Irvine, Irvine, CA, USA 92697-4560; 8 Department of Pharmaceutical Sciences, University of California, Irvine, Irvine, CA, USA 92697-4560

## Abstract

Administration of the Zeta Inhibitory Peptide (ZIP) interferes with memory maintenance and long-term potentiation (LTP). However, mice lacking its putative target, the protein kinase PKMζ, exhibit normal learning and memory as well as LTP, making ZIP’s mechanism unclear. Here, we show that ZIP disrupts LTP by removing surface AMPA receptors through its cationic charge alone. This effect was fully blocked by drugs that block macropinocytosis and is dependent on endophilin A2 (endoA2)-mediated endocytosis. ZIP and other cationic peptides selectively removed newly inserted AMPAR nanoclusters, providing a mechanism by which these peptides erase memories without effects on basal synaptic function. Lastly, cationic peptides can be administered locally and/or systemically and can be combined with local microinjection of macropinocytosis inhibitors to modulate memories on local and brain-wide scales. Our findings have critical implications for an entire field of memory mechanisms and highlight a previously unappreciated mechanism by which memories can be lost.

## INTRODUCTION:

A central question in neuroscience is how memories can persist for weeks to years even though proteins generally turn over in hours or days. One proposed solution to this conundrum is the existence of auto-feedback loops that create a self-sustaining signal that persists despite ongoing protein degradation. The discovery that injection of the zeta inhibitory peptide (ZIP), the autoinhibitory domain of the atypical protein kinase PKCζ, erased memories stored in the brain regions where ZIP was injected supported this idea^1^. This, and several follow-up studies, provided evidence in favor of the hypothesis that PKMζ, which lacks an autoinhibitory domain and is thus constitutively active, may be the kinase that facilitates such an auto-feedback loop essential for maintaining memories. However, two studies published in 2013 showed that 1) mice lacking the gene encoding PKMζ have normal learning and memory, and 2) injection of ZIP erases memories equally well in control and PKMζ knockout mice^2,3^, casting doubt on the PKMζ hypothesis. While the PKMζ hypothesis remains controversial^4–8^, it is still unclear what the actual mechanism is by which ZIP interferes with memory maintenance.

We noted that 6 of the 13 amino acids on ZIP are the cationic lysine or arginine. This is important given the long-known ability of cationic substances, including lipids and peptides, to induce endocytosis and act as delivery vehicles^9–12^. Several of these cationic delivery mechanisms enter cells through macropinocytosis^13,14^. Furthermore, ZIP is often administered at 10 mM^1,2,15^, a concentration at which non-specific, cationic interactions are likely to occur, stimulating endocytosis^10^. In this study, we tested the hypothesis that ZIP, at the concentrations typically used both *in vivo* (10 mM)^1,2,15^ and slice (1–5 μM)^3,15–17^, induces endocytosis of AMPARs non-specifically through cationic surface interactions.

## RESULTS:

### Cationic peptides cause endocytosis of surface AMPARs in a dose-dependent manner

While ZIP has largely been studied in the context of learning and memory, the TAT peptide has been broadly studied in cell biology for its use as a cell penetrating agent^18–21^. TAT is often attached to other peptides to facilitate cellular entry, which is mediated through its cationic charge. TAT shares no sequence similarity to ZIP, though both are highly cationic (ZIP sequence myr-SIY**RR**GA**RR**W**RK**L; TAT sequence G**RKKRR**Q**RRR**PQ). Many prior studies have also used scrambled ZIP (scrZIP) as a control (myr-**R**LY**RKR**IW**R**SAG**R**), with conflicting results; some indicate that ZIP and scrZIP have similar effects^3,5,22^, while others say that they do not^1,16,23,24^. If our hypothesis that charged peptides trigger endocytosis which then causes surface AMPAR removal is correct, then ZIP, scrZIP, TAT, and other cationic peptides should all mediate surface AMPAR removal. Furthermore, given that the cell-penetrating efficacy of cationic peptides is a function of charge magnitude^10,25^, we hypothesize that 1) the efficiency of AMPAR endocytosis should be peptide concentration-dependent, 2) other similarly cationic peptides should have a comparable ability as ZIP or TAT to stimulate AMPAR endocytosis, and 3) the effect of AMPAR endocytosis should scale with the magnitude of positive charge on the peptide.

**Figure 1:**
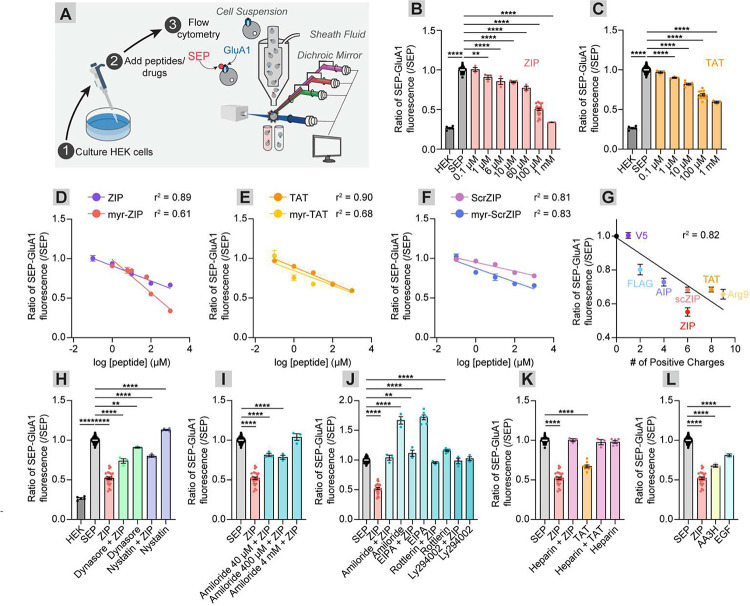
Cationic peptides trigger internalization of SEP-GluA1 in a charge- and dose-dependent manner. (A) Experimental schematic. Peptides or small molecules were added to confluent HEK SEP-GluA1 cells. Cellular fluorescence was assessed using flow cytometry. (B) ZIP application triggered internalization of SEP-GluA1 in a dose-dependent manner, with significant effects observed starting at 1 μM. The y-axis represents the mean of the FITC signal for the flow plot relative to that from untreated SEP-GluA1 cells. (C) TAT application triggered internalization of SEP-GluA1 in a dose-dependent manner, with significant effects observed starting at 1 μM. (D) Ratio of SEP-GluA1 internalization as a function of the log concentration of ZIP and myr-ZIP. (E) Ratio of SEP-GluA1 internalization as a function of the log concentration of TAT and myr-TAT. (F) Ratio of SEP-GluA1 internalization as a function of the log concentration of scrZIP and myr-scrZIP. (G) Ratio of SEP-GluA1 internalization as a function of the number of positive charges per peptide applied at 100 μM. (H) Dynasore (80 μM), chlorpromazine (5 μM) or nystatin (5 μg/μL) were applied to HEK SEP-GluA1 cells 4 hours prior to ZIP or vehicle application. Dynasore, chlorpromazine, and nystatin all partially but incompletely blocked ZIP’s effects. SEP vs. Dynasore/ZIP, 1.00 vs. 0.74, p < 0.0001, n = 114 and 3, respectively; ZIP vs. Dynasore/ZIP, 0.52 vs 0.74, p = 0.0002, n = 26 and 3, respectively; SEP vs. chlorpromazine/ZIP, 1.00 vs. 0.81, p < 0.0001, n = 114 and 3, respectively; ZIP vs. chlorpromazine/ZIP, 0.52 vs. 0.81, p < 0.0001; n = 26 and 3, respectively; SEP vs. nystatin/ZIP, 1.00 vs. 0.80, p < 0.0001, n = 114 and 3, respectively; ZIP vs. nystatin/ZIP, 0.52 vs. 0.80, p < 0.0001, n = 26 and 3, respectively. (I) Amiloride blocked ZIP’s effects at concentrations typically used to block macropinocytosis in cell culture, but not at lower concentrations. SEP vs. 40 μM amiloride/ZIP, 1.00 vs. 0.81, p < 0.0001, n = 114 and 3, respectively; SEP vs. 400 μM amiloride/ZIP, 1.00 vs. 0.79, p < 0.0001, n = 114 and 3, respectively; SEP vs. 4 mM amiloride/ZIP, 1.00 vs. 1.04, p = 0.51, n = 114 and 3, respectively. (J) Macropinocytosis inhibitors including amiloride, EIPA, rottlerin, and Ly294002 all completely blocked ZIP’s effects. SEP vs. ZIP/amiloride, 1.00 vs. 1.04, p = 0.86, n = 114 and 3, respectively; SEP vs. ZIP/EIPA, 1.00 vs 1.114, p = 0.029, n = 114 and 3, respectively; SEP vs ZIP/rottlerin, 1.00 vs 0.96, p = 0.85, n = 114 and 3, respectively; SEP vs. ZIP/Ly294002, 1.00 vs 0.99, p = 1.0, n = 114 and 3, respectively. (K) Neutralizing the charge of ZIP or TAT using heparin completely blocked their effects on SEP-GluA1 endocytosis. SEP vs. ZIP/heparin, 1.00 vs 0.98, p = 0.87, n = 114 and 6, respectively; TAT/heparin, 1.00 vs. 0.99, p = 1.0, n = 114 and 6, respectively. (L) Stimulating macropinocytosis using AA3H or EGF was able to significantly induce SEP-GluA1 endocytosis. SEP vs. EGF, 1.00 vs. 0.81, p < 0.0001, n = 114 and 3; SEP vs. AA3H, 1.00 vs. 0.68, p < 0.0001, n = 114 and 3, respectively.

To test these hypotheses, we first established an assay for measuring efficacy of AMPAR internalization utilizing HEK cells expressing super ecliptic pHluorin fused to the AMPAR subunit GluA1 (SEP-GluA1)^26^. SEP is a pH-sensitive GFP variant that is quenched in low-pH environments such as that found in endocytic vesicles^27^. Thus, SEP-GluA1 fluorescence reflects AMPARs at the cell surface and is useful for indirect quantitative measurements of endocytosis. SEP-GluA1-expressing HEK cells were treated with increasing concentrations of cationic peptide, gently released into suspension and subjected to flow cytometry to quantify changes in SEP-GluA1 surface fluorescence (Fig. 1A). Regarding hypothesis #1 that the efficiency of AMPAR endocytosis should be peptide concentration-dependent, application of ZIP stimulated a reduction in cellular fluorescence in a concentration-dependent manner (Fig. 1B), indicating that ZIP triggers endocytosis of SEP-GluA1. The same was observed for application of TAT (Fig. 1C).

One recent study hypothesized that ZIP’s function is dependent on the membrane docking function of its myristoyl group^28^. If ZIP’s cationic charge underlies its function, the myristoyl group should be dispensable. We are unaware of this having explicitly been tested in the literature. We tested the effects of ZIP, scrZIP, and TAT over a range of concentrations with and without the myristoyl group. ZIP, scrZIP, and TAT all induced removal of surface GluA1 in a concentration-dependent fashion, with or without the myristoyl group (Fig. 1D–F), though myr-ZIP was more potent at inducing AMPAR endocytosis at high concentrations (100 μM or higher), suggesting that other mechanisms beyond charge may participate at high concentrations (Fig. 1D). Notably, this effect was not observed with myr-TAT or myr-scrZIP (Fig. 1E–F).

Regarding hypothesis #2 that other similarly cationic peptides should have a similar ability to stimulate AMPAR endocytosis, we tested the effects of a variety of cationic and non-cationic peptides on AMPAR endocytosis. All cationic peptides that we tested had the same effect as ZIP or TAT on SEP-GluA1 endocytosis, including scrZIP, AIP (**KK**AL**RR**QEAVDAL), and Arg9 (**RRRRRRRRR**), but the non-cationic V5 (G**K**PIPNPLLGLDST) and FLAG (DY**K**DDDD**K**) peptides did not (Fig. 1G, S1A). Importantly, the effect of each peptide tested scaled positively with peptide concentration (Fig. S1B–D). Regarding hypothesis #3 that the effect on AMPAR endocytosis should scale with the number of positively charged residues on the peptide, we directly compared the ability of AIP (4 cationic residues, 31% of the peptide), ZIP and scrZIP (6 residues, 46% of the peptide), TAT (8 residues, 67% of the peptide) and Arg9 (9 residues, 100% of the peptide) to mediate the internalization of SEP-GluA1. The magnitude of AMPAR endocytosis scaled positively with the number of cationic charges of the peptide (linear regression, r^2^ = 0.82, p < 0.0001, Fig. 1G, S1F). These results together provide further evidence that ZIP’s function is mediated by its cationic charge.

### ZIP stimulates AMPAR internalization primarily through non-clathrin-mediated endocytosis

TAT is a model cell penetrating peptide typically appended to proteins of interest and can transport them into cells by triggering endocytosis, though the mechanism may be cargo-dependent^14,29–35^. To test which pathways are being utilized to trigger AMPAR endocytosis, we pre-incubated SEP-GluA1 HEK cells with a range of pathway-associated endocytosis modulators and assessed their effects on ZIP-induced AMPAR internalization. Preincubation with the dynamin inhibitor Dynasore or AP2 inhibitor chlorpromazine, which both interfere with clathrin-mediated endocytosis, or nystatin, which inhibits caveolin-mediated endocytosis, all significantly interfered with ZIP-mediated endocytosis of AMPARs (ZIP vs. Dynasore/ZIP, 0.52 vs 0.74, p = 0.0002, n = 26 and 3, respectively; ZIP vs. chlorpromazine/ZIP, 0.52 vs. 0.81, p < 0.0001; n = 26 and 3, respectively; ZIP vs. nystatin/ZIP, 0.52 vs. 0.80, p < 0.0001, n = 26 and 3, respectively; Fig. 1H, S2). However, the blockade was incomplete, indicating that ZIP likely uses other pathways, or perhaps a combination of pathways. Conversely, pre-incubation with amiloride, which blocks the class of sodium-proton antiporters (NHE) that mediate macropinocytosis, interfered with ZIP’s reduction in FITC signal in a dose-dependent fashion, with complete inhibition achieved by 4 mM, a concentration typically used to block macropinocytosis (SEP vs. ZIP/amiloride, 1.00 vs. 1.04, p = 0.08, n = 114 and 3, respectively)^10,30^ (Fig. 1I). As blocking NHEs would likely lead to endosomal hyperacidification^36–39^ which would quench fluorescence, the observed effects of amiloride are unlikely to be due to its effects on pH. Regardless, we tested several other macropinocytosis-inhibiting drugs including 5-(N-ethyl-N-isopropyl) amiloride (EIPA), rottlerin, and Ly294002, with the hypothesis that if all of these prevent ZIP-induced loss of fluorescence, then the effect is likely to be mediated by macropinocytosis. All three drugs prevented the ZIP-induced reduction in surface AMPARs, (SEP vs. ZIP/EIPA, 1.00 vs 1.11, p = 0.08, n = 114 and 3, respectively; SEP vs ZIP/rottlerin, 1.00 vs 0.9592, p = 0.98, n = 114 and 3, respectively; ZIP vs. ZIP/Ly294002, 1.00 vs 0.99, p = 1.0, n = 114 and 3, respectively) (Fig. 1J, S2). Therefore, macropinocytosis inhibitors are sufficient to completely block ZIP’s effect on AMPAR endocytosis, while inhibition of other forms of endocytosis only partially interferes with AMPAR endocytosis.

To further assess if ZIP works through its cationic charges alone, we tested if the anionic heparin could prevent ZIP’s effects through charge neutralization. Heparin was either pre-incubated with ZIP prior to administration to SEP-GluA1 cells or sequentially added to SEP-GluA1 cells immediately before the application of ZIP. Both methods eliminated ZIP and TAT’s ability to stimulate AMPAR endocytosis (SEP vs. ZIP/heparin, 1.00 vs 0.98, p = 0.87, n = 114 and 6, respectively; SEP vs. TAT/heparin, 1.00 vs. 0.99, p = 0.61, n = 114 and 6, respectively; Fig. 1K, S3A). Finally, use of non-cationic agents to stimulate macropinocytosis, such as the peptide epithelial growth factor (EGF) and a non-cationic cell-penetrating peptide derived from the membrane interacting protein annexin A, AA3H^40,41^ were sufficient to induce AMPAR endocytosis (SEP vs. EGF, 1.00 vs. 0.81, p < 0.0001, n = 114 and 3, respectively; SEP vs. AA3H, 1.00 vs. 0.68, p < 0.0001, n = 114 and 3, respectively) (Fig. 1L). Stimulating macropinocytosis is therefore both necessary and sufficient for ZIP-induced endocytosis of AMPARs in HEK cells.

### ZIP and TAT induce AMPAR endocytosis in neurons

**Figure 2:**
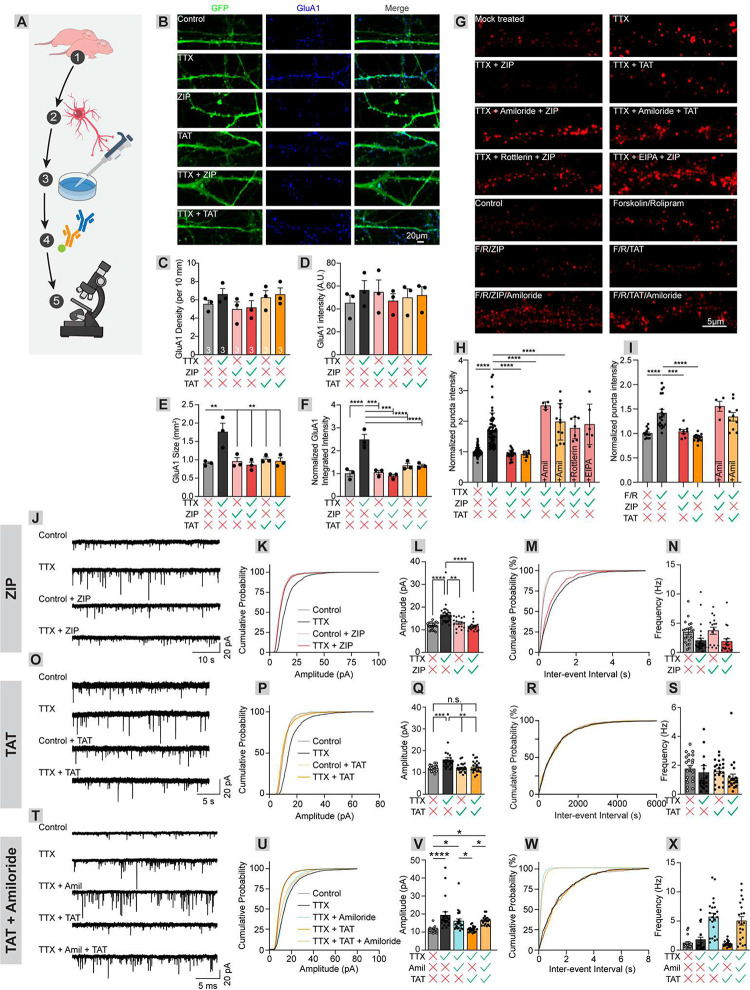
ZIP and TAT eliminate stimulus-induced elevations in GluA1, an effect blocked by amiloride. (A) Experimental schematic. Primary hippocampal cells were grown for 14 DIV. An elevation of surface AMPARs was then induced either through homeostatic plasticity via a two-day TTX application protocol, or forskolin + rolipram application. Small molecules and/or cationic peptides were then added to assess the effect on surface GluA1 levels. (B) Confocal micrograph of representative neuronal structures for each condition selected for quantitative analysis. A GFP-based cell-fill used to highlight dendritic structure is shown in green. GluA1 immunostaining is shown in blue. Scale bar, 20 μm. (C) Density of GluA1 puncta for each condition (per 10mm of dendrite) (Control vs. TTX+, 5.53 vs. 6.60, p = 0.88, n = 3 for both) (D) Mean intensity of GluA1 puncta for each condition (AU) (Control vs. TTX+, 45.25 vs. 56.35, p = 0.92, n = 3 for both) (E) Mean size of GluA1 puncta for each condition (mm^2^) (Control vs. TTX+, 0.90 vs 1.76, p = 0.003, n = 3 for both) (F) Normalized integrated puncta intensity for each condition (Control vs. TTX+, 1.00 vs 2.48, p < 0.0001, n = 3 for both; TTX vs. Control/ZIP, 2.48 vs. 1.05, p < 0.0001, n = 3 for both; TTX vs. TTX/ZIP, 2.48 vs. 0.90, p < 0.0001, n = 3 for both; TTX vs. Control/TAT, 2.48 vs. 1.35, p = 0.0006, n = 3 for both; TTX vs. TTX/TAT, 2.48 vs. 1.35, p = 0.0006, n = 3 for both). (G) Representative examples of confocal images for quantitative analysis of puncta intensity. Scale, 5 μm. (H) GluA1 levels after TTX application plus drugs and/or cationic peptides. TTX− vs. TTX+, 1.00 vs 1.63, p < 0.0001, n = 98 and 97, respectively; TTX− vs. TTX+/ZIP, 1.00 vs. 1.00, p = 1, n = 98 and 26, respectively; TTX− vs. TTX+ TAT, 1.00 vs. 0.92, p = 0.93, n = 98 and 7, respectively; TTX+ vs. TTX+/ZIP, 1.63 vs. 1.00, p < 0.0001, n = 97 and 26, respectively; TTX+ vs. TTX+/ZIP/40 μM amiloride 1.63 vs. 1.884, p = 0.92, n = 97 and 4, respectively; TTX+ vs. TTX+/ZIP/400 μM EIPA, 1.63 vs. 1.90, p = 0.81, n = 97 and 6, respectively; TTX+ vs. TTX+/ZIP/50 μM rottlerin, 1.63 vs. 1.78, p = 0.98, n = 97 and 7, respectively; TTX+ vs TTX+/TAT, 1.63 vs. 0.92, p = 0.002, n = 97 and 7, respectively; TTX+ vs. TTX+/TAT/4 mM amiloride, 1.63 vs. 1.98, p = 0.08, n = 97 and 12, respectively. (I) GluA1 levels after forskolin/rolipram application plus drugs and/or cationic peptides. Vehicle vs. forskolin/rolipram, 1.00 vs. 1.42, p < 0.0001, n = 14 and 19, respectively; vehicle vs. forskolin/rolipram/ZIP, 1.00 vs. 1.04, p = 0.99, n = 14 and 8, respectively; vehicle vs. forskolin/rolipram/ZIP, 1.00 vs. 0.91, p = 0.75, n = 14 and 14, respectively; forskolin/rolipram vs. forskolin/rolipram/ZIP/amiloride, 1.42 vs. 1.55, p = 0.72, n = 19 and 4, respectively; forskolin/rolipram vs. forskolin/rolipram/TAT/amiloride, 1.42 vs. 1.34, p = 0.86, n = 19 and 10, respectively. (J) Sample traces of miniature EPSCs recorded from control (no TTX), TTX-treated, no TTX + ZIP, and TTX + ZIP conditions. (K) Cumulative probability graph for EPSC amplitude for each condition. (L) Bar graph comparison of data shown in K. Control vs. TTX, 11.76 pA vs. 16.58 pA, p < 0.0001, n = 21 and 29, respectively; control vs. control/ZIP, 11.76 pA vs. 12.76 pA, p = 0.78, n = 21 and 16, respectively; control vs. TTX/ZIP, 11.76 pA vs. 11.56 pA, p = 1.00, n = 21 and 18, respectively; TTX vs. TTX/ZIP, 16.58 pA vs. 11.56 pA, p < 0.0001, n = 29 and 18, respectively. (M) Cumulative probability graph for the inter-event interval for each condition. (N) Bar graph comparison of EPSC frequency for each condition. Control vs. control/ZIP, 3.47 Hz vs. 3.72 Hz, p = 0.99, n = 21 and 16, respectively; control vs. TTX, 3.47 Hz vs. 2.00 Hz, p = 0.10, n = 21 and 29, respectively; TTX vs. TTX/ZIP, 2.00 Hz vs. 1.85 Hz, p = 1.00, n = 29 and 18, respectively; control/ZIP vs. TTX/ZIP, 3.72 Hz vs. 1.85 Hz, p = 0.069, n = 16 and 18, respectively. (O) Sample traces of miniature EPSCs recorded from control (no TTX), TTX-treated, no TTX + TAT, and TTX + TAT conditions. (P) Cumulative probability graph for EPSC amplitude for each condition. (Q) Bar graph comparison of data shown in P. Control vs. TTX, 11.88 pA vs. 14.91 pA, p < 0.0001, n = 21 and 23, respectively; control vs. TTX/TAT, 11.88 pA vs. 12.23 pA, p = 0.96, n = 21 and 23, respectively; TTX vs. TTX/TAT, 14.91 pA vs. 12.23 pA, p = 0.0005, n = 23 and 23, respectively. (R) Cumulative probability graph for the inter-event interval for each condition. (S) Bar graph comparison of EPSC frequency for each condition. Control vs. control/TAT, 1.79 Hz vs. 1.60 Hz, p = 0.97, n = 21 and 23, respectively; TTX vs. TTX/TAT, 1.56 Hz vs. 1.17 Hz, p = 0.76, n = 23 and 23, respectively. (T) Sample traces of miniature EPSCs recorded from control (no TTX), TTX-treated, TTX + amiloride, TTX + TAT, and TTX + TAT + amiloride conditions. (U) Cumulative probability graph for EPSC amplitude for each condition. (V) Bar graph comparison of data shown in U. Control vs. TTX, 11.34 pA vs. 19.37 pA, p < 0.0001, n = 19 and 21, respectively; TTX vs. TTX/TAT, 19.37 pA vs. 11.51 pA, p < 0.0001, n = 21 and 19, respectively; TTX vs. TTX/amiloride, 19.37 pA vs. 16.13 pA, p = 0.22, n = 21 and 22, respectively; TTX vs. TTX/TAT, 19.37 pA vs. 11.51 pA, p < 0.0001, n = 21 and 19, respectively; TTX vs. TTX/TAT/amiloride, 19.37 pA vs. 16.53 pA, p = 0.38, n = 21 and 19, respectively. (W) Cumulative probability graph for the inter-event interval for each condition. (X) Bar graph comparison of EPSC frequency for each condition. Control vs. TTX/amiloride, 1.23 Hz vs. 6.15 Hz, p < 0.0001, n = 21 and 23, respectively; Control vs. TTX/TAT/amiloride, 1.23 Hz vs. 4.36 Hz, p < 0.0001, n = 19 and 21, respectively.

To understand how ZIP may be impacting endogenous surface AMPARs on neurons, we need to use a neuronal system whereby endogenous surface AMPAR levels can be elevated by biological stimuli. To do this, we generated primary hippocampal cultures from postnatal day 0 mouse pups and grew them for 14 days *in vitro* (DIV) whereby they can form synaptic connections with other neurons (Fig. 2A)^42–44^. A primary hippocampal culture preparation is the ideal system to test the effects of cationic peptides because it is amenable to pharmacological manipulation and easily allows for the direct quantification of surface AMPARs.

We first utilized a well-studied neuronal silencing approach that elevates surface AMPARs through homeostatic plasticity^45^. To do this, we incubated the cultures for 48 hours in 500 nM tetrodotoxin (TTX), which blocks voltage-gated sodium channels. Prolonged treatment with TTX decreases neuronal excitability, triggering a compensatory up-scaling of synaptic AMPARs. We live surface labeled neuronal cultures with GluA1 antibodies to label only surface-exposed AMPARs, then fixed and applied secondary antibodies and imaged using confocal microscopy^46^ (Fig. 2A). We first tested whether TTX could induce long-lasting changes in GluA1 density, intensity, size, or a combination of these factors. TTX induced a significant elevation in puncta size that was reversed by ZIP or TAT application, but no change in any other metrics (GluA1 puncta size, Control vs. TTX+, 0.90 vs 1.76, p = 0.003, n = 3 for both; GluA1 puncta density, Control vs. TTX+, 5.53 vs. 6.60, p = 0.88, n = 3 for both; GluA1 puncta intensity, Control vs. TTX+, 45.25 vs. 56.35, p = 0.92, n = 3 for both; Fig. 2B–E). The intensity of surface GluA1 puncta per dendrite length (integrated intensity of surface GluA1 divided by dendrite length (in pixels)), normalized to control was used hereafter (Control vs. TTX+, 1.00 vs 2.48, p < 0.0001, n = 3 for both; TTX vs. TTX/ZIP, 2.48 vs. 0.90, p < 0.0001, n = 3 for both; TTX vs. TTX/TAT, 2.48 vs. 1.35, p = 0.0006, n = 3 for both; Fig. 2F). Importantly, we did not observe an effect of ZIP or TAT application on the basal number of surface AMPARs (control vs. Control/ZIP, 0.90 vs. 0.95, p 1.0, n = 3 for both; control vs. Control/TAT, 0.90 vs. 1.02, p = 0.98, n = 3 for both; Fig. 2B–F), indicating that ZIP or TAT do not impact constitutively present synaptic AMPARs but rather induce endocytosis specifically of AMPARs inserted following induction of plasticity.

Next, we assessed the effects of macropinocytosis inhibitors on ZIP- and TAT-mediated reduction in surface AMPARs. As before, relative to mock treated neurons, TTX robustly elevated surface AMPAR levels as observed by a ~1.6-fold elevation in the average intensity of GluA1 puncta (TTX− vs. TTX+, 1.00 vs 1.63, p < 0.0001, n = 98 and 97, respectively; Fig. 2H) that was completely reversed by a 10 minute application of 100 μM ZIP or TAT immediately before immunostaining (TTX− vs. TTX+/ZIP, 1.00 vs. 1.00, p = 1, n = 98 and 26, respectively; TTX− vs. TTX+ TAT, 1.00 vs. 0.92, p = 0.93, n = 98 and 7, respectively; Fig. 2H). This ZIP-induced reduction in surface AMPARs was blocked by prior application of amiloride, EIPA, or rottlerin (TTX+ vs. TTX+/ZIP, 1.63 vs. 1.00, p < 0.0001, n = 97 and 26, respectively; TTX+ vs. TTX+/ZIP/40 μM amiloride 1.63 vs. 1.884, p = 0.92, n = 97 and 4, respectively; TTX+ vs. TTX+/ZIP/400 μM EIPA, 1.63 vs. 1.90, p = 0.81, n = 97 and 6, respectively; TTX+ vs. TTX+/ZIP/50 μM rottlerin, 1.63 vs. 1.78, p = 0.98, n = 97 and 7, respectively; Fig. 2H). Application of amiloride also prevented the TAT-induced reduction in surface AMPARs (TTX+ vs TTX+/TAT, 1.63 vs. 0.92, p = 0.002, n = 97 and 7, respectively; TTX+ vs. TTX+/TAT/4 mM amiloride, 1.63 vs. 1.98, p = 0.08, n = 97 and 12, respectively; Fig. 2H). To test if this mechanism is relevant to other forms of plasticity-induced elevations of surface AMPARs, we used 50 μM forskolin, an adenylyl cyclase enzyme activator, and 0.1 μM rolipram, a phosphodiesterase-4 inhibitor, which together rapidly increase synaptic cAMP levels in post-synaptic neurons to generate a stable potentiation-like state in magnesium-deficient media^47^. Forskolin and rolipram treatment elevated surface AMPAR levels (vehicle vs. forskolin/rolipram, 1.00 vs. 1.42, p < 0.0001, n = 14 and 19, respectively) (Fig. 2I) which were eliminated by ZIP or TAT application (vehicle vs. forskolin/rolipram/ZIP, 1.00 vs. 1.04, p = 0.99, n = 14 and 8, respectively; vehicle vs. forskolin/rolipram/ZIP, 1.00 vs. 0.91, p = 0.75, n = 14 and 14, respectively) and this reduction was in turn prevented by prior application of amiloride (forskolin/rolipram vs. forskolin/rolipram/ZIP/amiloride, 1.42 vs. 1.55, p = 0.72, n = 19 and 4, respectively; forskolin/rolipram vs. forskolin/rolipram/TAT/amiloride, 1.42 vs. 1.34, p = 0.86, n = 19 and 10, respectively) (Fig. 2I). ZIP and TAT thus reduce surface AMPARs on neurons via mechanisms consistent with macropinocytosis.

### Electrophysiological evidence of ZIP and TAT’s effects on AMPAR-mediated transmission

The above assays do not indicate whether cationic effects impact synaptic transmission. To test this, we performed whole-cell patch clamp recordings on primary hippocampal neurons following TTX-mediated homeostatic plasticity and/or application of ZIP, TAT, and amiloride. TTX induced an elevation in the amplitude of miniature excitatory post-synaptic currents (EPSCs; control vs. TTX, 11.76 pA vs. 16.58 pA, p < 0.0001, n = 21 and 29, respectively; Fig. 2J–L). ZIP had no effect on mEPSC amplitude in non-TTX-treated cultures, but reduced TTX-mediated elevations in mEPSC amplitude to baseline (control vs. control/ZIP, 11.76 pA vs. 13.01 pA, p = 0.78, n = 21 and 16, respectively; control vs. TTX/ZIP, 11.76 pA vs. 11.56 pA, p = 1.00, n = 21 and 18, respectively; TTX vs. TTX/ZIP, 16.58 pA vs. 11.56 pA, p < 0.0001, n = 29 and 18, respectively) (Fig. 2J–L). These results, combined with our morphological analyses, indicate that ZIP administration does not reduce basal levels of surface or synaptic AMPARs. Importantly, ZIP did not influence mEPSC frequency (control vs. control/ZIP, 3.47 Hz vs. 3.72 Hz, p = 0.99, n = 21 and 16, respectively; TTX vs. TTX/ZIP, 2.00 Hz vs. 1.85 Hz, p = 1.00, n = 29 and 18, respectively) (Fig. 2M–N). The effect of TAT application was similar to ZIP, with TAT application having no effect on baseline AMPAR transmission but reducing TTX-mediated elevations in mEPSC amplitude to baseline (control vs. TTX, 12.00 pA vs. 15.82 pA, p = 0.0007, n = 22 and 24, respectively; control vs. TTX/TAT, 12.00 pA vs. 12.47 pA, p = 0.96, n = 22 and 24, respectively; TTX vs. TTX/TAT, 15.82 pA vs. 12.47 pA, p = 0.0027, n = 24 and 24, respectively) (Fig. 2O–Q). Like ZIP, no effect of TAT application was observed on mEPSC frequency (control vs. control/TAT, 1.79 Hz vs. 1.60 Hz, p = 0.97, n = 21 and 23, respectively; TTX vs. TTX/TAT, 1.537 Hz vs. 1.17 Hz, p = 0.76, n = 24 and 23, respectively) (Fig. 2R–S). Notably, TTX, ZIP, or TAT did not affect membrane resistance, capacitance, or mEPSC kinetics (Fig. S4). Amiloride application alone did not have any effect on mEPSC amplitude (TTX vs. TTX/amiloride, 19.37 pA vs. 16.13 pA, p = 0.22, n = 21 and 22, respectively), but prevented the TAT-induced reduction in mEPSC amplitude (TTX vs. TTX/TAT, 19.37 pA vs. 11.51 pA, p < 0.0001, n = 21 and 19, respectively; TTX vs. TTX/TAT/amiloride, 19.37 pA vs. 16.53 pA, p = 0.38, n = 21 and 19, respectively) (Fig. 2T–V). Interestingly, amiloride application elevated mEPSC frequency (TTX vs. TTX/amiloride, 1.80 Hz vs. 6.15 Hz, p < 0.0001, n = 21 and 23, respectively) (Fig. 2W–X). These results together support our conclusions that ZIP or TAT application reduces synaptic AMPAR levels, and this effect can be rescued by prior amiloride administration.

### ZIP and TAT eliminate newly inserted AMPAR nanoclusters with minimal effect on basal AMPARs

**Figure 3:**
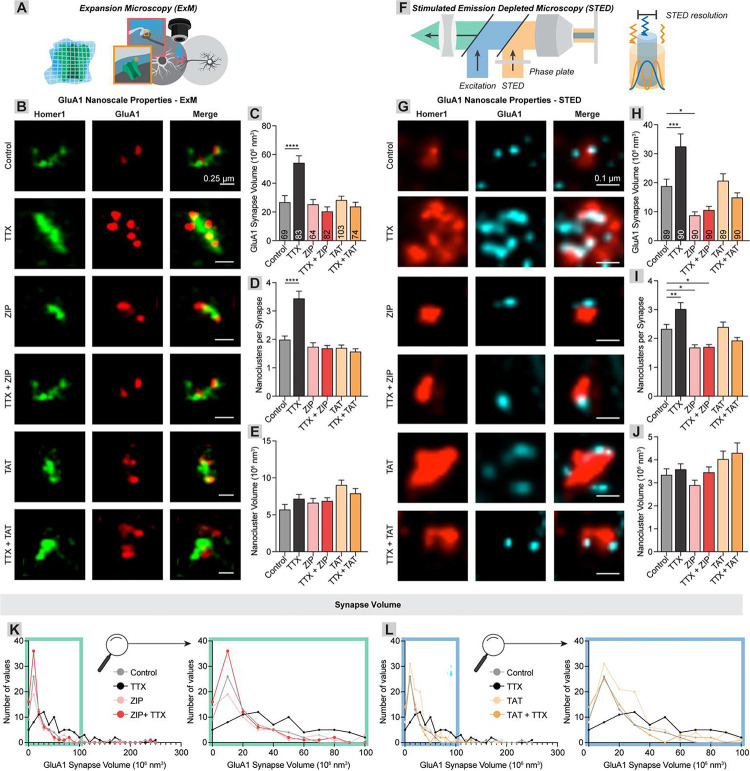
ZIP and TAT remove newly inserted AMPAR nanoclusters with no impact on AMPAR nanocluster volume. (A) Schematic of Expansion Microscopy. (B) Sample Expansion Microscopy confocal images of Homer1, GluA1, and merged for control, TTX, ZIP, TTX + ZIP, TAT, TTX + TAT. (C) Total volume occupied by GluA1 in the synapse (control vs. TTX, 26.7 vs. 54.1, p < 0.0001, n = 69 and 83; control vs. ZIP, 26.7 vs. 25.2, p = 1.0, n = 69 and 64; control vs. TTX/ZIP, 26.7 vs. 20.2, p = 0.64, n = 69 and 81; control vs. TAT, 26.7 vs. 28.2, p = 1.0, n = 69 and 103; control vs. TTX/TAT, 26.7 vs. 23.7, p = 0.98, n = 69 and 74). (D) Numbers of GluA1 nanoclusters per synapse (control vs. TTX, 1.99 vs. 3.43, p < 0.0001, n = 69 and 83; control vs. ZIP, 1.99 vs. 1.73, p = 0.73, n = 69 and 64; control vs. TTX/ZIP, 1.99 vs. 1.68, p = 0.52, n = 69 and 81; control vs. TAT, 1.99 vs. 1.70, p = 0.53, n = 69 and 103; control vs. TTX/TAT, 1.99 vs. 1.56, p = 0.23, n = 69 and 74). (E) Volume of GluA1 nanoclusters (control vs. TTX, 5.70 vs. 7.14, p = 0.38, n = 69 and 83; control vs. ZIP, 5.70 vs. 6.61, p = 0.81, n = 69 and 64; control vs. TTX/ZIP, 5.70 vs. 6.84, p = 0.60, n = 69 and 81; control vs. TAT, 5.70 vs. 9.00, p = 0.001, n = 69 and 103; control vs. TTX/TAT, 5.70 vs. 7.89, p = 0.09, n = 69 and 74). (F) Schematic of STED microscopy. (G) Sample STED microscopy images of Homer1, GluA1, and merged for control, TTX, ZIP, TTX + ZIP, TAT, TTX + TAT. (H) Total volume occupied by GluA1 in the synapse (control vs. TTX, 18.7 vs. 32.4, p = 0.0006, n = 89 and 90; control vs. ZIP, 18.7 vs. 8.7, p = 0.02, n = 89 and 90; control vs. TTX/ZIP, 18.7 vs. 10.5, p = 0.08, n = 89 and 90; control vs. TAT, 26.7 vs. 20.6, p = 0.98, n = 89 and 89; control vs. TTX/TAT, 18.7 vs. 14.8, p = 0.70, n = 89 and 90). (I) Numbers of GluA1 nanoclusters per synapse (control vs. TTX, 2.33 vs. 3.01, p = 0.0078; n = 89 and 90; control vs. ZIP, 2.33 vs. 1.68, p = 0.014, n = 89 and 90; control vs. TTX/ZIP, 2.33 vs. 1.70, p = 0.019, n = 89 and 90; control vs. TAT, 2.33 vs. 2.39, p = 1.0, n = 89 and 89; control vs. TTX/TAT, 2.33 vs. 1.92, p = 0.23, n = 89 and 90). (J) Volume of GluA1 nanoclusters (control vs. TTX, 3.36 vs. 3.58, p = 0.97; control vs. ZIP, 3.36 vs. 2.89, p = 0.76; control vs. TTX/ZIP, 3.36 vs. 3.45, p = 1.0; control vs. TAT, 3.36 vs. 4.02, p = 0.37; control vs. TTX/TAT, 3.36 vs. 4.29, p = 0.11; n = 90 for all). (K) Histogram of GluA1 volume in the synapse for control, TTX, ZIP, and TTX/ZIP conditions. (L) Histogram of GluA1 volume in the synapse for control, TTX, TAT, and TTX/TAT conditions.

The development of super-resolution imaging approaches has revealed that proteins essential for synapse function are assembled into regions of high density. These high-density regions are commonly termed “nanoclusters” and are thought to be critical for synaptic transmission. Given the proposed importance of synaptic nanoarchitecture, we next assessed ZIP and TAT’s effects on the synaptic nanostructure. ZIP reverses LTP with minimal to moderate effects on basal synaptic function^48–51^, suggesting that mostly LTP-induced AMPARs are affected. To investigate this question, we used two different methods of super-resolution microscopy to identify individual AMPAR nanoclusters within synapses. First, we expanded the tissue ~3.6 times using Expansion Microscopy and imaged using a confocal microscope (Fig. 3A). TTX-induced homeostatic plasticity elevated the total GluA1 volume within the synapse and increased the number of synaptic GluA1 nanoclusters without a corresponding increase in the volume of each nanocluster (Synaptic GluA1 volume: control vs. TTX, 26.7 vs. 54.1, p < 0.0001; nanocluster number: control vs. TTX, 1.99 vs. 3.43, p < 0.0001; nanocluster volume: control vs. TTX, 5.70 vs. 7.14, p = 0.38, n = 69 and 83 for all; Fig. 3A–E). ZIP and TAT reduced synaptic GluA1 volume and the number of nanoclusters to baseline, with no effect on baseline levels (Synaptic GluA1 volume: control vs. ZIP, 26.7 vs. 25.2, p = 1.0, n = 69 and 64; control vs. TTX/ZIP, 26.7 vs. 20.2, p = 0.64, n = 69 and 81; control vs. TAT, 26.7 vs. 28.2, p = 1.0, n = 69 and 103; control vs. TTX/TAT, 26.7 vs. 23.7, p = 0.98, n = 69 and 74; nanocluster number: control vs. ZIP, 1.99 vs. 1.73, p = 0.73, n = 69 and 64; control vs. TTX/ZIP, 1.99 vs. 1.68, p = 0.52, n = 69 and 81; control vs. TAT, 1.99 vs. 1.70, p = 0.53, n = 69 and 103; control vs. TTX/TAT, 1.99 vs. 1.56, p = 0.23, n = 69 and 74; Fig. 3A–E).

To complement the expansion microscopy approach, we used STimulated Emission-Depletion (STED) super-resolution microscopy to measure the same properties in non-expanded tissue (Fig. 3F). Again, TTX increased the total synaptic GluA1 volume within the synapse through an increase in the number of nanoclusters, with no change in nanocluster volume (Synaptic GluA1 volume: control vs. TTX, 18.7 vs. 32.4, p = 0.0006; nanocluster number: control vs. TTX, 2.33 vs. 3.01, p = 0.0078; n = 89 and 90 for both, Fig. 3G–J). The number, but not volume, of nanoclusters was reduced to ~baseline, with a slight reduction in ZIP-treated cultures (Synaptic GluA1 volume: control vs. ZIP, 18.7 vs. 8.7, p = 0.02, n = 89 and 90; control vs. TTX/ZIP, 18.7 vs. 10.5, p = 0.08, n = 89 and 90; control vs. TAT, 26.7 vs. 20.6, p = 0.98, n = 89 and 89; control vs. TTX/TAT, 18.7 vs. 14.8, p = 0.70, n = 89 and 90; nanocluster number: control vs. ZIP, 2.33 vs. 1.68, p = 0.014, n = 89 and 90; control vs. TTX/ZIP, 2.33 vs. 1.70, p = 0.019, n = 89 and 90; control vs. TAT, 2.33 vs. 2.39, p = 1.0, n = 89 and 89; control vs. TTX/TAT, 2.33 vs. 1.92, p = 0.23, n = 89 and 90; Fig. 3G–J).

While cationic peptides reduce nanocluster numbers to baseline, this could occur either due to a uniform reduction in nanoclusters, or preferential reduction in homeostatic plasticity-induced nanoclusters. To differentiate these possibilities, we investigated the distribution of GluA1 synapse volumes across synapses. TTX shifted the distribution to the right, indicating formation of high GluA1 volume synapses at the expense of low volume synapses (Fig. 3K–L), presumably through the insertion of new AMPARs and formation of new nanoclusters. The right shifted redistribution of GluA1 volume via the incorporation of new AMPARs is consistent with previous findings^52^ and with the accepted underlying mechanism of TTX-induced homeostatic plasticity. The ZIP- and TAT-induced reduction of these synaptic volumes could be due to an ~equal removal of recently inserted AMPARs and recently formed AMPAR nanoclusters from all synapses, which would be indicated by an overall suppression of the distribution, or preferential removal of newly inserted nanoclusters, which would manifest as a leftward shift of the curve. Both ZIP and TAT shifted the distribution to the left, similar to baseline conditions, indicating that ZIP and TAT preferentially remove newly inserted AMPAR nanoclusters (Fig. 3K–L).

### Cationic peptides trigger synaptic AMPAR removal through Endophilin A2-mediated endocytosis

**Figure 4:**
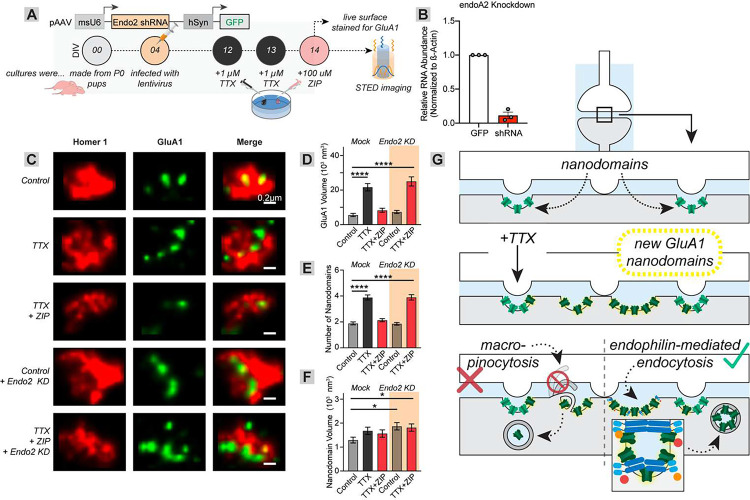
ZIP’s removal of newly inserted AMPAR nanoclusters is dependent on Endophilin-A2. (A) Experimental schematic and timeline. (B) shRNA-mediated knockdown efficiency of EndoA2 mRNA. (C) Sample STED microscopy images of Homer1, GluA1, and merged for control, TTX, TTX + ZIP, shRNA, shRNA + TTX + ZIP. (D) Total volume occupied by GluA1 in the synapse (control vs. TTX, 5.58 vs 21.64, p < 0.0001; control vs. TTX/ZIP, 5.58 vs. 8.23, p = 0.63; control vs. shRNA, 5.58 vs. 7.32, p = 0.88; control vs. shRNA/TTX/ZIP, 5.58 vs. 25.02, p < 0.0001; n = 90 for all). (E) Numbers of GluA1 nanoclusters per synapse (control vs. TTX, 1.89 vs. 3.89, p < 0.0001; control vs. TTX/ZIP, 1.89 vs. 2.13, p = 0.64; control vs. shRNA, 1.89 vs. 1.86, p = 1.0; control vs. shRNA/TTX/ZIP, 1.89 vs. 3.90, p < 0.0001; n = 90 for all). (F) Volume of GluA1 nanoclusters (control vs. TTX, 1.29 vs. 1.68, p = 0.19; control vs. TTX/ZIP, 1.29 vs. 1.57, p = 0.49; control vs. shRNA, 1.29 vs. 1.87, p = 0.02; control vs. shRNA/TTX/ZIP, 1.29 vs. 1.81, p = 0.04; n = 90 for all). (G) Schematic for hypothesized mechanism of action. TTX induces homeostatic plasticity, largely through increasing the number of nanoclusters. Cationic peptides trigger remodeling of the membrane through EndoA2-mediated endocytosis, which is activated only upon cationic peptide-mediated stimulation. This preferentially removes newly inserted AMPAR nanodomains.

Our data are all consistent with cationic peptides mediating synaptic AMPAR removal through macropinocytosis. However, macropinocytosis is unlikely to take place at the synapse, because the synaptic cleft is approximately 20–30 nm while macropinocytosis involves a 200 nm – 6 μm membrane protrusion and formation of macropinosomes with a diameter 10–200x the size of the neuronal synapse^53–56^. As clathrin- and caveolin-mediated endocytosis were not sufficient to fully block ZIP and TAT’s effects (Fig. 1–2), we tested if endophilin A2 (endoA2)-mediated endocytosis^57^ may be responsible. To test this, we introduced an endoA2 shRNA into primary neuronal cultures at DIV4 and tested the effects of ZIP on TTX-induced elevations in the nanoscale properties of AMPARs, imaged using STED (Fig. 4A–B). TTX once again elevated the volume of GluA1 within the synapse and number of GluA1 nanoclusters with no significant effect on nanocluster volume, and ZIP reduced these to baseline (GluA1 synapse volume: control vs. TTX, 5.58 vs 21.6, p < 0.0001; control vs. TTX/ZIP, 5.58 vs. 8.28, p = 0.63; GluA1 nanocluster number: control vs. TTX, 1.89 vs. 3.89, p < 0.0001; control vs. TTX/ZIP, 1.89 vs. 2.13, p = 0.64, n = 90 each for all comparisons, Fig. 4C–F). Expression of the endoA2 shRNA alone did not alter the basal the nanoscopic properties of GluA1, but it prevented the ZIP-induced reduction of the TTX-induced elevation in the synaptic GluA1 volume and number of GluA1 nanoclusters (GluA1 synapse volume: control vs. shRNA, 5.58 vs. 7.32, p = 0.88; control vs. shRNA/TTX/ZIP, 5.58 vs. 25.02, p < 0.0001; GluA1 nanocluster number: control vs. shRNA, 1.89 vs. 1.86, p = 1.0; control vs. shRNA/TTX/ZIP, 1.89 vs. 3.90, p < 0.0001, n = 90 each for all comparisons, Fig. 4C–F). Thus, while macropinocytosis-blocking drugs can prevent cationic peptide-induced reduction in synaptic AMPARs, the effect in neurons is mediated by endoA2 (Fig. 4G).

### Stimulation of non-clathrin-mediated endocytosis is necessary and sufficient for ZIP’s effects on memory

**Figure 5:**
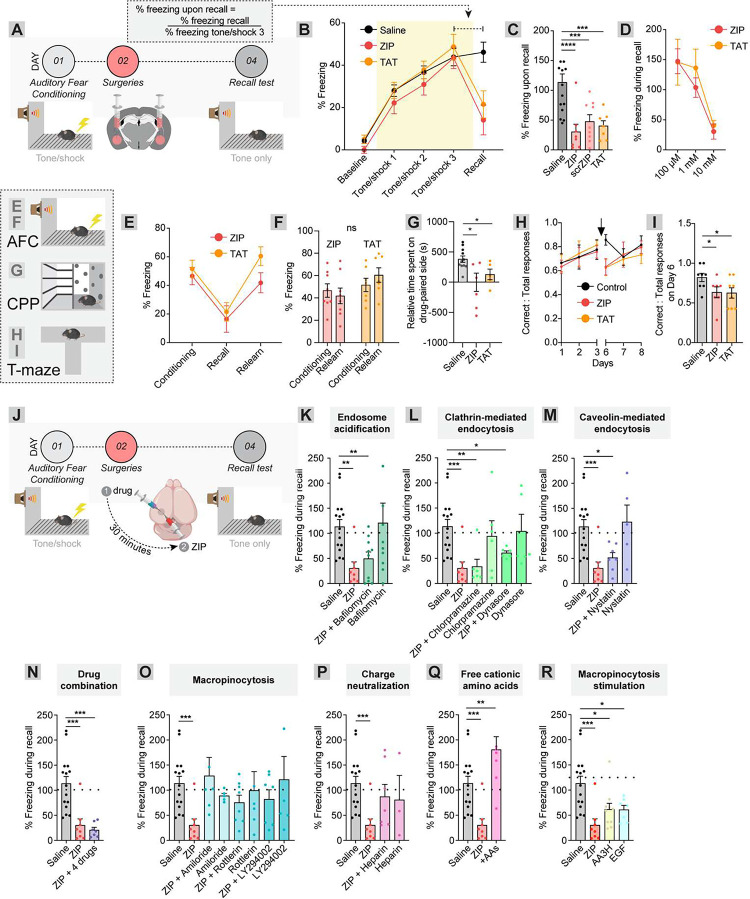
Local injection of cationic peptides erases memories stored near the site of injection, an effect blocked by macropinocytosis inhibitors (A) Experimental schematic. Mice were trained in an auditory fear conditioning (AFC) task where they learn to associate a neutral tone (conditioned stimulus) with an aversive foot shock (unconditioned stimulus). The following day, ZIP, scrZIP, or TAT were injected into the BLA. After one full day for recovery, mice were then placed back into the chamber, and the time spent freezing during tone presentation was assessed. (B) Time spent freezing during the tone alone as well as tone-shock pairings on Day 1, and the tone presentation alone (Recall) on Day 4. The % freezing upon recall is the ratio of the percentage of time spent freezing during the recall test relative to the final tone/shock association. (C) While saline infusion had no effect on recall, infusion of ZIP, scrZIP, and TAT strongly impaired recall of the tone/shock association. p < 0.0001, two-way ANOVA; Saline vs. ZIP, average normalized freezing 113.4 vs. 30.35, p = 0.001, n = 15 saline, n = 8; saline vs. scrZIP, average normalized freezing 113.4 vs. 47.81, p = 0.0009, n = 15 saline, n = 9 scrZIP; saline vs. TAT, average normalized freezing 113.4 vs. 40.41, p = 0.0007, n = 15 saline, n = 7 TAT. (D) This effect was concentration-dependent, as concentrations lower than 10 mM peptide had an insignificant effect. (E) ZIP or TAT infusion impaired recall of the tone/shock association, and animals were able to re-learn this association equally well as during the first learning period. (F) Comparison of the time spent freezing the third tone/shock association before and after ZIP or TAT administration. ZIP, conditioning 46.59, relearn 41.82, p = 0.61, n = 8; TAT conditioning average 51.57, relearn 60.47, p = 0.33, n = 7. (G) Both ZIP and TAT impaired recall of the conditioned place preference memory. Saline vs. ZIP, time spent on cocaine-paired side 381.8 vs. −1.5 seconds, p = 0.012, n = 11 saline, n = 7 ZIP; saline vs. TAT, time spent on cocaine-paired side 381.8 vs 129 seconds, p = 0.022, n = 11 saline, n = 5 TAT. (H) ZIP or TAT injection impaired recall of learned spontaneous alternation. Saline Day 6 vs. Day 3, 0.86 vs. 0.78, p = 0.41, n = 8; ZIP Day 6 vs. Day 3, 0.63 vs. 0.77, p = 0.03, n = 7; TAT Day 6 vs. Day 3, 0.58 vs. 0.80, p = 0.04, n = 8. (I) ZIP or TAT injection into the dorsal hippocampus impaired recall of learned spontaneous alternation when tested on day 6. Control vs ZIP, 0.82 vs. 0.63, p = 0.04, n = 8 and 7, respectively; Control vs. TAT, 0.82 vs. 0.63, p = 0.03, n = 8 for both. (J) Experimental schematic. Experiments were performed as in Fig. 5A, except that a small molecule was administered into the BLA 30 minutes prior to ZIP application. (K) Bafilomycin administration had no effect on ZIP-mediated memory disruption. ZIP vs. ZIP/bafilomycin, 30.35% vs. 64.19%, p = 0.14, n = 8 and 5, respectively. (L) Clathrin-mediated endocytosis inhibitors chlorpromazine and Dynasore only mildly impaired ZIP-mediated memory disruption. ZIP vs. ZIP/chlorpromazine, 30.35% vs. 33.54%, p = 0.87, n = 8 and 6, respectively; ZIP vs. ZIP/Dynasore, 30.35% vs. 60.89%, p = 0.07, n = 8 and 6, respectively. (M) The caveolin-mediated endocytosis inhibitor nystatin only mildly impaired ZIP-mediated memory disruption. ZIP vs. ZIP/nystatin, 30.35% vs. 51.64%, p = 0.22, n = 8 and 7, respectively. (N) A combination of bafilomycin, chlorpromazine, Dynasore, and nystatin had no effect on ZIP-mediated memory disruption. ZIP vs. 4 drug cocktail, 30.35% vs. 20.76%, p = 0.52, n = 8 and 7, respectively. (O) Administration of amiloride, rottlerin, and Ly294002 all blocked ZIP-mediated memory disruption. Saline vs. ZIP/amiloride, 113.4% vs. 128.6%, p = 0.64, n = 15 and 6, respectively; saline vs. rottlerin/ZIP, 113.4% vs. 75.48%, p = 0.10, n = 15 and 8, respectively; saline vs. Ly294002/ZIP, 113.4% vs. 81.88%, p = 0.22, n = 15 and 7, respectively. (P) Heparin administration completely blocked ZIP-mediated memory disruption. Saline vs. ZIP/heparin, 113.4% vs. 87.19%, p = 0.34, n = 15 and 7, respectively. (Q) Administration of positively charged amino acids did not disrupt recall of the tone/shock association. Saline vs. AAs, 113.4% vs. 180.5%, p = 0.02, n = 15 and 8, respectively. (R) Stimulating macropinocytosis via AA3H or EGF application impaired recall of the tone/shock association. Saline vs. EGF, 113.4% vs. 61.17%, p = 0.03, n = 15 and 7, respectively; saline vs. AA3H, 113.4% vs. 61.68, p = 0.02, n = 15 and 10, respectively.

We compared the consequences of administering ZIP, scrZIP, or TAT on memory in mice, using auditory fear conditioning as a model of behavioral plasticity. In this task, mice learn to associate a previously neutral tone (conditioned stimulus) with an aversive foot shock (unconditioned stimulus), and subsequently demonstrate freezing behavior upon presentation of the conditioned stimulus alone. The basolateral amygdala encodes the associative memory, at least for several days post-training, whereafter it is believed to be gradually transferred to the prefrontal cortex^58–63^. Administration of ZIP into the BLA has previously been shown to eliminate memories of the tone-shock association^16,22^. Consistent with these findings, injection of 1 μL of 10 mM ZIP bilaterally into the BLA 24 hours after conditioning was sufficient to significantly impair memory of the tone-shock association, as assayed by reduced freezing following tone presentation (saline vs. ZIP, average normalized freezing 113.4 vs. 30.35, p = 0.001, n = 15 saline, n = 8 ZIP; Fig. 5A–C). Infusion of an equal volume/concentration of scrZIP or TAT had a similar effect on memory recall, while infusion of saline had no effect (p < 0.0001, two-way ANOVA; saline vs. scrZIP, average freezing 113.4 vs. 47.81, p = 0.0009, n = 15 saline, n = 9 scrZIP; saline vs. TAT, average freezing 113.4 vs. 40.41, p = 0.0007, n = 15 saline, n = 7 TAT; Fig. 5C). ZIP and TAT were only effective in disrupting auditory fear conditioning memories when infused at a concentration of 10 mM, as lower concentrations had little to no effect on memory persistence (Fig. 5D). Infusion of ZIP or TAT did not cause long-term damage to the BLA, as mice could re-learn the tone/shock association upon re-training (ZIP, conditioning 46.59, relearn 41.82, p = 0.61, n = 8; TAT conditioning average 51.57, relearn 60.47, p = 0.33, n = 7; Fig. 5E–F). Like in HEK SEP-GluA1 cells, the efficacy of peptides in impairing memory recall scaled linearly with the number of positive charges on the peptides (Fig. S5).

If ZIP and TAT work through disrupting surface AMPAR stability, they should be able to disrupt other memories that are encoded by elevations in synaptic AMPAR levels, such as cocaine conditioned place preference, which leads to an elevation in surface AMPARs in the nucleus accumbens (NAc)^64,65^. Relative to mice infused with 1 μL of saline, infusion of 1 μL of 10 mM ZIP into the NAc eliminated expression of cocaine place preference, as demonstrated previously^66,67^ (saline vs. ZIP, time spent on cocaine-paired side 381.8 vs. −1.5 seconds, p = 0.012, n = 11 saline, n = 7 ZIP, Fig. 5G). TAT also eliminated place preference (saline vs. TAT, time spent on cocaine-paired side 381.8 vs 129 seconds, p = 0.022, n = 11 saline, n = 5 TAT, Fig. 5G). We also tested the effect of ZIP or TAT on a hippocampus-based learning task, learned spontaneous alternation. In this task, the amount of spontaneous alternation in maze navigation is assessed each day. Mice improve their performance over time, increasing the amount of correct spontaneous alternations (Fig. 5H–I), which is linked to AMPAR function^68–70^. After showing improvement over 3 days of learning, ZIP or TAT were infused into the dorsal hippocampus. The percentage of correct responses upon re-testing in ZIP- and TAT-treated mice dropped to baseline levels, whereas animals that received saline infusions continued performing with a higher ratio of correct responses (Saline Day 6 vs. Day 3, 0.86 vs. 0.78, p = 0.41, n = 8; ZIP Day 6 vs. Day 3, 0.63 vs. 0.77, p = 0.03, n = 7; TAT Day 6 vs. Day 3, 0.58 vs. 0.80, p = 0.04, n = 8; Fig. 5I). This is consistent with other results indicating ZIP infusion into the dorsal hippocampus disrupts spatial memory^16^, and indicates that infusion of TAT (and scrZIP) can recapitulate the memory disrupting effects of ZIP.

To assess ZIP’s mechanism of action *in vivo*, we performed auditory fear conditioning experiments, although this time instead of injecting only ZIP on day 2, we preceded ZIP administration by an injection of a drug to block different types of endocytosis 30 minutes prior (Fig. 5J). Inhibition of endocytic maturation (Fig. 5K), clathrin-mediated endocytosis (Fig. 5L), caveolin-mediated endocytosis (Fig. 5M), or a combination of these processes (Fig. 5N) had no significant effect on ZIP’s ability to erase auditory fear conditioning memories (ZIP vs. ZIP/bafilomycin, 30.35% vs. 64.19%, p = 0.14, n = 8 and 5, respectively; ZIP vs. ZIP/chlorpromazine, 30.35% vs. 33.54%, p = 0.87, n = 8 and 6, respectively; ZIP vs. ZIP/Dynasore, 30.35% vs. 60.89%, p = 0.07, n = 8 and 6, respectively; ZIP vs. ZIP/nystatin, 30.35% vs. 51.64%, p = 0.22, n = 8 and 7, respectively; ZIP vs. 4 drug cocktail, 30.35% vs. 20.76%, p = 0.52, n = 8 and 7, respectively). These results mirror those found in the SEP-GluA1 HEK cells (Fig. 1). In contrast, prior administration of macropinocytosis inhibitors prevented ZIP’s ability to interfere with auditory fear recall (saline vs. ZIP/amiloride, 113.4% vs. 128.6%, p = 0.64, n = 15 and 6, respectively; saline vs. rottlerin/ZIP, 113.4% vs. 75.48%, p = 0.10, n = 15 and 8, respectively; saline vs. Ly294002/ZIP, 113.4% vs. 81.88%, p = 0.22, n = 15 and 7, respectively) (Fig. 5O), as did neutralization of ZIP’s charge through prior administration of heparin (saline vs. ZIP/heparin, 113.4% vs. 87.19%, p = 0.34, n = 15 and 7, respectively) (Fig. 5P). The high charge density of ZIP was necessary for ZIP’s effects, as administration of the equivalent amount of free cationic amino acids (50 mM arginine plus 10 mM lysine) did not recapitulate ZIP’s effects; in fact, it potentiated recall (saline vs. amino acids (AAs), 113.4% vs. 180.5%, p = 0.02, n = 15 and 8, respectively) (Fig. 5Q). These results indicate that stimulating macropinocytosis is necessary for ZIP to erase memories *in vivo*. We then tested if administration of macropinocytisis-triggering agents could interfere with memory. Both EGF and AA3H impaired auditory fear recall (saline vs. EGF, 113.4% vs. 61.17%, p = 0.03, n = 15 and 7, respectively; saline vs. AA3H, 113.4% vs. 61.68, p = 0.02, n = 15 and 10, respectively) (Fig. 5R). Thus, stimulating the biological pathways underlying macropinocytosis is both necessary and sufficient for disrupting auditory fear conditioning memories.

### Cationic peptides and inhibitors enable systemic and local modulation of memories

While ZIP is typically administered locally in the brain, evidence suggests that cell penetrating peptides such as TAT can enter the brain when administered peripherally^14^. While there is some controversy as to whether any peptide functionally enters cells throughout the body following systemic administration^71–74^, our data suggest that stimulating non-clathrin-mediated endocytosis alone is sufficient to mediate ZIP and TAT’s effects. Thus, even if much of the peptide attaches locally to vasculature, and/or does not get released from endosomes and enters cells, we still expect to see effects on memory.

**Figure 6:**
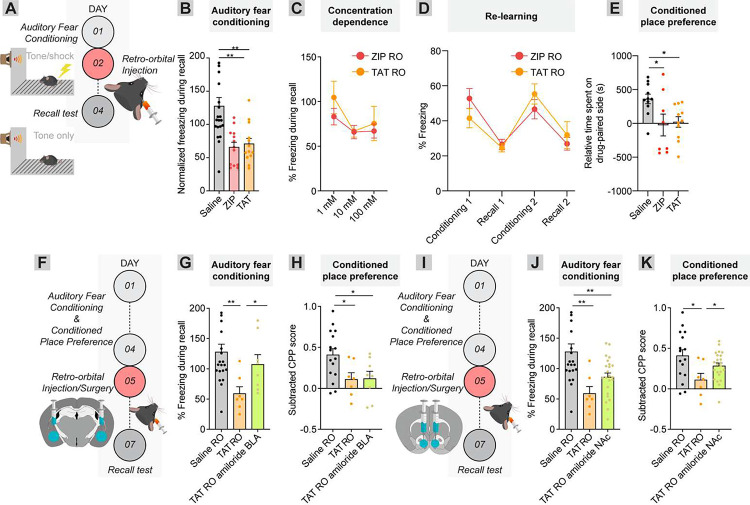
Local and global memory modulation by ZIP and amiloride. (A) Experimental schematic of retroorbital administration of cationic peptides. (B) Retroorbital administration of either ZIP or TAT significantly impaired recall of the tone/shock association. Saline vs. ZIP, 127.8% vs. 65.88%, p = 0.0011, n = 21 and 13, respectively; saline vs. TAT, 127.8% vs. 70.87, p = 0.0027, n = 21 and 13, respectively. (C) Impairment of the tone/shock association was concentration-dependent, with at least 10 mM being required to observe a significant effect. Saline vs. 1 mM ZIP, 127.8% vs. 89.39%, p = 0.10, n = 21 and 7, respectively; saline vs. 1 mM TAT, 127.8% vs. 104.6%, p = 0.33, n = 21 and 8, respectively; ZIP 10 mM vs. ZIP 100 mM, 65.88% vs. 67.07%, p = 0.93, n = 13 and 5, respectively; TAT 10 mM vs. TAT 100 mM, 66.76% vs. 75.45%, p = 0.59, n = 20 and 8, respectively. (D) Mice could re-learn the tone/shock association after retroorbital administration, and ZIP or TAT could be repeatedly injected with a similar effect on the tone/shock association each time. ZIP, recall 1 vs. conditioning 2, 26.57% vs. 46.66%, p = 0.0048, n = 10; TAT, recall 1 vs. conditioning 2, 24.54% vs. 55.32%, p < 0.0001, n = 10; ZIP, conditioning 2 vs. recall 2, 46.66% vs. 26.92%, p = 0.0081, n = 10; TAT, conditioning 2 vs. recall 2, 55.32% vs. 31.89%, p 0.02, n = 10. (E) Retroorbital injection of ZIP or TAT interfered with recall of conditioned place preference. Saline vs. ZIP, 0.34 vs. −0.03, p = 0.06, n = 16 and 6, respectively; saline vs. TAT, 0.34 vs. 0.11, p = 0.09, n = 16 and 7, respectively. (F) Schematic of retroorbital TAT administration preceded by local infusion of amiloride into the BLA. (G) This perturbation had no effect on the tone/shock association. Saline RO vs. TAT RO, 127.8% vs. 59.13%, p = 0.0091, n = 21 and 7, respectively; saline RO vs. TAT RO/amiloride BLA, 127.8% vs. 107.4%, p = 0.57, n = 21 and 8, respectively. (H) This perturbation resulted in a significant loss of the conditioned place preference memory. Saline RO vs. TAT RO, 0.41 vs. 0.11, p = 0.041, n = 16 and 7, respectively; saline RO vs. TAT RO/amiloride BLA, 0.41 vs. 0.12, p = 0.038, n = 16 and 8, respectively. (I) Schematic of retroorbital TAT administration preceded by local infusion of amiloride into the NAc. (J) This perturbation resulted in a significant impairment of the tone/shock association. Saline RO vs. TAT RO/amiloride NAc, 127.8% vs. 86.02%, p = 0.0068, n = 21 and 23, respectively. (K) This perturbation had no effect on the conditioned place preference memory. Saline RO vs. TAT RO/amiloride BLA, 0.41 vs. 0.28, p = 0.18, n = 16 and 23, respectively.

To test this, we first injected 10 mM ZIP or TAT retro-orbitally one day following auditory fear conditioning and tested the effect on recall (Fig. 6A). Administration of 1 μL of 10 mM ZIP or TAT impaired memory (saline vs. ZIP, 127.8% vs. 65.88%, p = 0.0011, n = 21 and 13, respectively; saline vs. TAT, 127.8% vs. 70.87, p = 0.0027, n = 21 and 13, respectively) (Fig. 6B). This effect was milder than direct intracranial injection into the BLA (saline vs. ZIP into the BLA, 113.4% freezing vs. 30.4%; saline vs. ZIP retroorbital injection, 127.8% freezing vs. 65.9%), and was concentration-dependent, as no significant effect was observed on memory following administration of 1 mM of ZIP or TAT (saline vs. ZIP, 127.8% vs. 89.39%, p = 0.10, n = 21 and 7, respectively; saline vs. TAT, 127.8% vs. 104.6%, p = 0.33, n = 21 and 8, respectively); 100 mM peptide had no further effect beyond 10 mM (ZIP 10 mM vs. ZIP 100 mM, 65.88% vs. 67.07%, p = 0.93, n = 13 and 5, respectively; TAT 10 mM vs. TAT 100 mM, 66.76% vs. 75.45%, p = 0.59, n = 20 and 8, respectively) (Fig. 6C). Systemic administration of these peptides did not cause long-term impairment in brain function, as mice could re-learn the tone-shock association after cationic peptide administration (ZIP, recall 1 vs. conditioning 2, 26.57% vs. 46.66%, p = 0.0048, n = 10; TAT, recall 1 vs. conditioning 2, 24.54% vs. 55.32%, p < 0.0001, n = 10), and subsequent systemic administration of peptide again impaired memory (ZIP, conditioning 2 vs. recall 2, 46.66% vs. 26.92%, p = 0.0081, n = 10; TAT, conditioning 2 vs. recall 2, 55.32% vs. 31.89%, p 0.02, n = 10) (Fig. 6D). In addition, systemic delivery of ZIP or TAT also impaired conditioned place preference, thought to be stored in the NAc (saline vs. ZIP, 361.4s vs. −23.6s, p = 0.03, n = 11 and 8, respectively; saline vs. TAT, 361.4s vs. 20.9s, p = 0.03, n = 11 and 1, respectively) (Fig. 6E). Therefore, systemic administration of cationic peptides can interfere with memories stored throughout the brain.

We showed that amiloride can prevent ZIP or TAT’s effects (Fig. 1, 2, 5). Here, rather than locally interfering with a memory where ZIP or TAT is injected, we instead injected TAT systemically, and amiloride intracranially into a brain region where we wanted a memory *preserved*. We then trained animals to acquire both an auditory fear conditioning memory, stored in the BLA, and a cocaine conditioned place preference memory, stored in the NAc. Following learning of both associations, we injected amiloride into either the NAc or BLA, and injected TAT retro-orbitally 30 minutes later. When TAT was injected retro-orbitally and amiloride injected into the BLA, the conditioned place preference memory was impaired while the auditory fear conditioning memory was intact (fear conditioning, saline RO vs. TAT RO, 127.8% vs. 59.13%, p = 0.0091, n = 21 and 7, respectively; saline RO vs. TAT RO/amiloride BLA, 127.8% vs. 107.4%, p = 0.57, n = 21 and 8, respectively; conditioned place preference, saline RO vs. TAT RO, 0.41 vs. 0.11, p = 0.041, n = 16 and 7, respectively; saline RO vs. TAT RO/amiloride BLA, 0.41 vs. 0.12, p = 0.038, n = 16 and 8, respectively) (Fig. 6F–H). When TAT was injected retro-orbitally and amiloride was injected into the NAc, the auditory fear conditioning memory was impaired, while the conditioned place preference memory was intact (fear conditioning, saline RO vs. TAT RO/amiloride NAc, 127.8% vs. 86.02%, p = 0.0068, n = 21 and 23, respectively; conditioned place preference, saline RO vs. TAT RO/amiloride BLA, 0.41 vs. 0.28, p = 0.18, n = 16 and 23, respectively) (Fig. 6I–K). Therefore, cationic peptides can be used in combination with amiloride to modulate memory retention on both local and global scales.

## DISCUSSION:

Here we show that ZIP impairs memory not through any specific molecular interaction, but rather through general cationic surface-mediated initiation of endocytosis. This agrees with several previous studies that PKMζ is not required^2,3^; in fact, it is possible that no protein targets are required, but rather that the interaction with negatively charged cellular membranes is sufficient^75,76^. Our results using drugs that impair various stages of endocytosis and endosome maturation such as bafilomycin (Fig. 5K) indicate that ZIP likely doesn’t have to get into cells at all; rather, stimulation of endocytosis is sufficient, as this process likely removes AMPARs from the surface. While a recent manuscript reported that ZIP works through arginine metabolism, here we show that stimulating non-clathrin-mediated endocytosis through endoA2 is responsible, though other endocytosis mechanisms may contribute^77,78^. Furthermore, we found that 1) the myristoyl group isn’t required for ZIP’s function, 2) administering ZIP or an equal concentration of free lysines and arginines yield different results, 3) clathrin-mediated endocytosis plays only a minor role in ZIP’s effects on AMPAR endocytosis, all of which contrast to the previous study. We also show for the first time that TAT administration systemically and amiloride injection locally is sufficient to maintain memories specifically at the site of amiloride administration. While it is commonly assumed that synaptic processes such as LTP underlie memories, except for a few cases^79,80^, the evidence directly linking LTP/long-term depression (LTD) and memories is sparse. Our data provide additional support for the hypothesis that excitatory plasticity at a defined brain site mediates memories by showing that amiloride, which prevents cationic peptide-induced loss of memories, can preserve memories stored in the BLA for auditory fear conditioning, and the NAc for conditioned place preference.

Our study also adds to a growing body of literature indicating that ZIP and scrZIP have similar effects on memory and LTP. While it is possible that the different order of amino acids in these peptides affects peptide charge density which may in turn influence interactions with the cellular membrane and the mechanisms of endocytosis engaged^81^ (scrambled ZIP is more diffuse), we expect this difference to be minimal. Notably, previous studies showed that application of 100nM of the full-length HIV TAT protein interferes with LTP in acute slice preparations, and a version lacking the domain that includes the cationic peptide used in this study had no effect on LTP^82^. While macropinocytosis inhibitors blocked ZIP and TAT’s effects on surface AMPARs, given the small space in the synapse we believe that endoA2, a key player in fast endophilin-mediated endocytosis (FEME), is responsible. While endoA2’s role in ultrafast endocytosis in pre-synaptic terminals has been characterized^57,83^, endoA2 also localizes to post-synaptic membranes and directly interacts with GluA1, regulating its endocytosis^84–89^. Notably, FEME is not constitutively active and therefore likely does not contribute to processes related to basal AMPAR levels, but rather is triggered upon stimulation via ligands, here the cationic peptides, to remove synaptic AMPARs.

Mounting evidence supports the notion that within synapses, proteins critical for synaptic transmission are non-uniformly distributed and assemble into highly enriched nanoclusters^90–93^. Indeed, AMPARs form postsynaptic nanoclusters that align with presynaptic RIM1, which has been proposed to facilitate action-potential evoked synaptic transmission while spontaneous mEPSCs may be regulated by AMPARs that do not participate in nanoclusters^91,94,95^. While there is consensus that new AMPARs are required to strengthen synapses in response to LTP- and homeostatic plasticity-inducing stimuli, to our knowledge, the properties of these new AMPARs at the nanoscale level remain largely untested. Previous work demonstrated that TTX-induced homeostatic plasticity increases the nanoscale abundance of AMPARs and PSD95^52,96^, and our data reveal that in addition to increased synaptic AMPARs, the number of AMPAR nanoclusters are enhanced following TTX treatment. While we cannot exclude the possibility that new AMPARs also incorporate into preexisting nanoclusters, one intriguing mechanism proposed is that new AMPARs diffuse into the postsynaptic density and create new nanoclusters^97^. These new synaptic AMPARs can be clustered by phosphatidylinositol-(3,4,5)-trisphosphate (PIP_3_). The basal abundance of PIP_3_ is maintained at low concentrations, however, it can be rapidly produced following the induction of LTP and homeostatic plasticity and is enriched in excitatory synapses^98–101^. In addition to clustering AMPARs, PIP_3_ and its subsequent dephosphorylation to PIP_2_ mediates the recruitment of endophilins to the membrane^102^. This mechanism, identified in non-neuronal cells, represents an attractive explanation why newly trafficked AMPARs, induced by TTX, are more sensitive to FEME. Thus, elevated levels of PIP_3_ may be a mechanism that both clusters new AMPARs to help form new nanoclusters while also making these nanoclusters sensitive to removal.

One seeming conundrum is that all the inhibitors used in this study that block clathrin-dependent endocytosis (Dynasore, AP2) or clathrin-independent endocytosis (amiloride, nystatin) reduce or prevent ZIP- and TAT-mediated endocytosis of AMPARs, which raises the mechanistically unsatisfying possibility that ZIP and TAT may indiscriminately induce endocytosis. While we cannot completely exclude the contributions of non-FEME processes of endocytosis, FEME is sensitive to all inhibitors tested here^102^. Further, knockdown of endoA2 specifically inhibits FEME and does not alter clathrin-mediated endocytosis or macropinocytosis^103,104^. Thus, our direct manipulation of endoA2 expression strongly supports the notion that FEME primarily mediates the removal of AMPARs induced by ZIP and TAT.

Importantly, many studies have used TAT as an intracellular delivery vehicle for small peptides. Several of these studies report findings consistent with a loss of memories via loss of surface AMPARs^105–107^. Further, the cationic AIP peptide has been used as an inhibitor of CamKII^108^, and its effects when added to acute slice preparations mimics that of ZIP and other cationic peptides^109^. While it is possible that the mechanism of AIP-induced AMPAR endocytosis occurs through CaMKII inhibition, several studies indicate that this may not be how AIP exerts its effects. For example, AIP administration into the brain typically occurs at mM concentrations^110^, much like ZIP; at these concentrations, cationic effects are likely to play an important role. Notably, AIP has been shown to interact with CaMKII via cationic effects^111^, and AIP is not specific for CamKII^112^. Given these results, it is possible that cationic surface interactions mediated by TAT and AIP may be influencing the observed results and suggest that caution should be taken when interpreting data from experiments using cationic peptides.

## Methods:

### Mice:

Mice were housed on a 12-hour light–dark cycle with food and water *ad libitum*. Males and females from the C57/BL6 background were used for all behavioral experiments and for primary neuronal culture experiments in approximately equal proportions. All surgeries were done under isoflurane anesthesia. All procedures complied with the animal care standards set forth by the National Institute of Health and were approved by the Institutional Animal Care and Use Committees (IACUC) at the University of California, Irvine and University of Colorado Anschutz Medical Campus. All primary neuronal culture procedures were conducted in accordance with guidelines approved by Administrative Panel on Laboratory Animal Care at University of Colorado, Anschutz School of Medicine, accredited by Association for Assessment and Accreditation of Laboratory Animal Care International (AAALAC; 00235).

### Peptides:

All peptides were purchased from Peptide2.0. Peptides were dissolved at a stock concentration of 100 mM in 0.9% saline; dilutions were then made of these stocks into saline for use as working stocks.

**Table:** 

Peptide name	Sequence (+/−)	Net charge (pH	MW (kDa)
ZIP	SIY**RR**GA**RR**W**RK**L	+6	1.718
myr-ZIP	myr-SIY**RR**GA**RR**W**RK**L	+6	1.928
scrZIP	**R**LY**RKR**IW**R**SAG**R**	+6	1.718
myr-scrZIP	myr-**R**LY**RKR**IW**R**SAG**R**	+6	1.928
TAT	G**RKKRR**Q**RRR**PQ	+8	1.621
myr-TAT	myr-G**RKKRR**Q**RRR**PQ	+8	1.831
AA3H	MASIWVG**HR**G	+1.09	1.113
AA3H-PLP	MASIWVG**HR**G-**RRR**QQQQQQ**RRR**	+7.09	2.819
V5	G**K**PIPNPLLGLDST	0	1.421
FLAG	DY**K**DDDD**K**	−3	1.012
Arg9	**RRRRRRRRR**	+9	1.423
AIP	myr-**KK**AL**RR**QEAVDAL	+2	1.708

### Chemicals and concentrations:

The chemicals and peptides in this manuscript were used in the concentrations listed below:

Dynasore: *in vitro*, 8 μM, 80 μM; *in vivo*, 50 μM

chlorpromazine: *in vitro*, 0.5 μM, 5 μM; *in vivo*, 5 μg/mL

nystatin: *in vitro*, 0.05 μg/mL, 0.5 μg/mL, 5 μg/mL; *in vivo*, 50 nM

Ly294002: *in vitro*, 0.5 μM, 5 μM, 50 μM; *in vivo*, 100 μM

rottlerin: *in vitro*, 0.5 μM, 5 μM, 50 μM; *in vivo*, 50 μM

amiloride: *in vitro*, 40 μM, 400 μM, 4 mM; *in vivo*, 4 mM

EIPA: *in vitro*, 4 μM, 40 μM, 400 μM

bafilomycin: *in vitro*, 0.1 μM, 1 μM; *in vivo*, 1 μM

heparin: *in vitro*, 20 μg/mL; *in vivo*, 20 μg/mL

EGF: *in vitro*, 1 μM; *in vivo*, 1 μM

AA3H: *in vitro,* 10 μM, 100 μM; *in vivo*, 10 mM

AA3H-PLP: *in vitro*, 10 μM, 100 μM

*myr*-ZIP: *in vitro,* 0.1 μM, 1 μM, 6 μM, 10 μM, 60 μM, 100 μM, 1 mM; *in vivo*, 100 μM, 1 mM, 10 mM, 100 mM

ZIP (no myr): *in vitro*, 0.1 μM, 1 μM, 10 μM, 100 μM, 1 mM

TAT: *in vitro,* 0.1 μM, 1 μM, 10 μM, 100 μM, 1 mM; *in vivo*, 100 μM, 1 mM, 10 mM, 100 mM

*myr*-TAT: *in vitro,* 0.1 μM, 1 μM, 10 μM, 100 μM, 1 mM

scrZIP: *in vitro,* 0.1 μM, 1 μM, 10 μM, 100 μM, 1 mM; *in vivo*, 10 mM

scrZIP (no myr): *in vitro,* 0.1 μM, 1 μM, 10 μM, 100 μM, 1 mM

AIP: *in vitro,* 100 μM

V5: *in vitro,* 100 μM; *in vivo*, 10 mM

FLAG: *in vitro,* 100 μM; *in vivo*, 10 mM

Arg9: *in vitro,* 100 μM; *in vivo*, 10 mM

**Table:** 

REAGENT	MANUFACTURER	CATALOGUE #
DMEM-HG	Gibco (Thermo-Fisher)	11995073
FBS	Gibco (Thermo-Fisher)	FB12999102
TrypLE	Gibco (Thermo-Fisher)	12605010
Penicillin-Streptomycin	Gibco (Thermo-Fisher)	15140122
Sodium Citrate	Fisher Scientific	BP327-500
Sodium Chloride	Fisher Scientific	NC0377033
Potassium Chloride	Sigma	P9541
Bovine Serum Albumin fraction	Fisher Scientific	BP1600100
PBS	Gibco (Thermo-Fisher)	70011044
Dynasore	Fisher Scientific	501873184
Nystatin	Sigma	N6261
Amiloride	Fisher Scientific	AAJ6216803
EIPA	Sigma	A3085-25MG
Rottlerin	ABCAM	ab120377
Ly294002	Cell Signaling	9901S
Heparin	Fisher Scientific	2812100
EGF	Sigma	E9644
V5 peptide	Sigma	V7754
FLAG peptide	Sigma	F3290
Bafilomycin	Fisher Scientific	50464897
Forskolin	Fisher Scientific	F0855-10MG
Rolipram	Fisher Scientific	50-593-0
Chlorpromazine HCl	Sigma	C8138
α-GluA1	EMD Millipore	MAB2263MI
Donkey α-Mouse Alexa 647	Jackson Immunoresearch	AB_2340862

### SEP-GluA1 cell culture assays:

HEK SEP-GluA1 cells were obtained as a gift from Dr. Paul Temkin. These cells express the rat GluA1 with an N-terminus fusion of SEP^113^. HEK cells not expressing SEP-GluA1 were used as controls. Cell populations were maintained in Dulbecco’s Minimum Essential Media - High Glucose (Gibco) supplemented with 10% fetal bovine serum (Gibco) and 1x penicillin/streptomycin (Gibco) and passaged at 70–80% confluence using TrypLE (Gibco) per ATCC recommendations for HEK293T cells. Cells were plated for analysis in 6-well plates 48 hours prior to use, at the ATCC-recommended confluence (150,000–200,000 cells/well).

All conditions performed in triplicate. Except as otherwise described in time course assays, endocytosis modulators were added to wells 4 hours prior to experimentation. As necessary, modulators were stored dry in −20C or kept dissolved in DMSO solutions and stored at −20C in stocks of high enough concentration sufficient to yield a final DMSO concentration ≤ 0.1% v/v. Treatment peptides/chemicals were added to wells and allowed to incubate for 10 minutes. After incubation, the treatment media was removed and replaced with 1.5 mL ice-cold pH 7.0 citrate-buffered saline (135mM NaCl, 2.68mM KCl, 35.7mM Na_3_C_6_H_5_O_7_,) and all well plates were placed on ice for 5 minutes of incubation. Cells were gently detached with sterile cell scrapers, gently triturated with a P1000 pipette, and transferred to FACS tubes preloaded with 1.5 mL iced 0.5% BSA-PBS. FACS tubes were centrifuged at 170 rcf for 5 minutes at 4C, and all supernatant was removed. Cells were gently resuspended in 300 μL iced 0.5% BSA-PBS and tubes packed in ice for transport.

Assistance with flow cytometry was provided by the UCI Institute for Immunology Flow Cytometry Facility, a shared resource supported by the Chao Family Comprehensive Cancer Center. Samples were analyzed on the ACEA NovoCyte Quanteon at 50 μL/min on the 488 nm/530 nm laser/detector pairing, recording 200,000 events or 60 seconds. Data analysis was performed using FlowJo^™^ v10.8 Software (BD Life Sciences). Analyses were gated by forward- vs. side-scatter to exclude debris and clusters per accepted practice, then by side-scatter height vs. area to isolate intact single HEK cells; following that, whole samples were analyzed for arithmetic mean FITC signal, and the means were normalized to untreated SEP-HEK-GluA1.

### Cultured hippocampal Neurons:

Primary hippocampal cultures were prepared as previously described^46^ . Briefly, P0-P1 mice were sacrificed, and hippocampi were isolated from the brain in Hank’s Buffered Salt Solution (HBSS) with careful attention to avoid meningeal or cortical contamination. Hippocampi were digested in 10 U/ml Papain in HBSS at 37C for 20 minutes. Isolated hippocampi were then washed once with Plating Media (Earle’s MEM, 2 mM Glutamine, 0.4% Glucose, 5% fetal bovine serum, 1:50 Gem21 NeuroPlex) and triturated with a 1000μL pipette 15–20 times. Dissociated neurons were then plated onto sonicated, and acid stripped #1.5 coverslips (Warner Instruments Cat# 64–0714) previously coated with Matrigel^®^ Basement Membrane Matrix (Corning Cat# 356237) at a density of 200,000 cells per well of a 24 well plate or 400,000 cells per well of a 12 well plate with subsequent gentle agitation to ensure an even distribution of neurons on the coverslip. On DIV1, 70% of the media was changed with Growth Media (Earle’s MEM, 2.38 mM Sodium Bicarbonate, 2 mM Glutamine, 0.4% Glucose, 0.1 mg/ml apo-transferrin, 5% Fetal bovine serum, 1:50 Gem21 NeuroPlex). On DIV3, when a glial monolayer had developed, 70% of the media was exchanged with Growth Media supplemented to achieve a final concentration of 4 μM Cytosine β-D-arabinofuranoside (AraC; Sigma Cat# C1768) to arrest glial cell growth. Homeostatic potentiation is induced on neurons by addition of TTX (Abcam), to a final concentration of 500 μM, on DIV14 and DIV15. Measurements are performed on DIV16. For forskolin/rolipram assays, TTX was not added, and for parity cultures were permitted to grow until DIV16. For LTP induction, neuronal growth media was replaced with magnesium-free induction ACSF (in mM: 120 NaCl, 26 NaHCO_4_, 1 NaH_2_PO_4_, 2.5 KCl, 2.5 CaCl_2_, and 10 D-glucose) supplemented with 50 μM forskolin (Fisher Cat# F0855) and 100 nM rolipram (Fisher Cat# 50–593-0) and incubated at 37C for 5 minutes. Following that, media was replaced with magnesium-supplemented induction ACSF (1.3 mM MgSO_4_) and incubated for 30 minutes prior to application of peptide. 400 μM amiloride was added to magnesium-supplemented induction ACSF prior to incubation for amiloride treatment groups.

### Generation of endophilin-A2 shRNA:

shRNA directed against endogenous mouse endoA2 was generated as previously described^93^. We used previously validated target sequences for mouse endoA2^114^: 5’ GAACCTGTGTGACAAGGAT 3’. The target sequence was inserted downstream of a human U6 promoter between Hpa/XhoI restriction sites in a lentivirus transfer vector using the following primers Forward: 5’ TGAACCTGTGTGACAAGGATTTCAAGAGAATCCTTGTCACACAGGTTCTTTTTTC 3’ and Reverse 5’ TCGAGAAAAAAGAACCTGTGTGACAAGGATTCTCTTGAAATCCTTGTCACACAGGTTCA 3’. The lentivirus transfer plasmid also contained a human synapsin promoter driving eGFP. Lentiviruses were produced as previously described in HEK293T cells^93,115^. Briefly, cells were transfected with the lentivirus transfer vector, pMDL gag/pol, pRSV Rev and pCMV VSV-G using the calcium phosphate method. 18 hrs after transfection, transfection efficiency was assessed by the presence of GFP fluorescence. Cells were washed 3x with PBS and incubated with neuron growth medium. 24hrs after the addition of growth medium, the media was harvested and centrifuged for 5 min at 1500×g to pellet cell debris. Lentivirus was either used immediately or aliquoted and stored at −80 C. ~80 μl of viral supernatant was added to each well. Infection efficiency was determined by neuron fluorescence.

### Quantification of shRNA knockdown:

shRNA knockdown of endogenous mouse endophilin-A2 in neurons was assessed by RT-qPCR using predesigned primers and probes from Integrated DNA Technologies as previously described^93^. Primer 1: 5’ TGACATCCACCTTCTTTTCCA 3’; Primer 2: 5’ GAAGAAGCAGTTCTACAAGGC 3’; Probe: CCACCAACCTTCTCGCTGACCA. mRNA from primary mouse hippocampal neurons infected with control or endoA2 shRNA lentiviruses was harvested on DIV 14–16 with a Quick-RNA micro-prep mRNA isolation kit (Zymo research, Cat#: R1050). The concentration of mRNA was normalized between samples. mRNA was quantified using qScript XLT 1-Step RT-qPCR ToughMix kit (Quanta-bio, Cat#: 95132–100) on a Bio-Rad CFX384 Real-Time qPCR system. The resulting values were normalized to β-actin using pre-designed primers (Integrated DNA Technologies) Primer 1: GACTCATCGTACTCCTGCTTG, Primer 2: GATTACTGCTCTGGCTCCTAG, Probe: CTGGCCTCACTGTCCACCTTCC.

### Immunofluorescence:

For live surface-labeling of GluA1, primary hippocampal neurons were washed 1x with pre-warmed HEPES-based ACSF, which contained (in mM): 140 NaCl, 5 KCl, 2.5 CaCl2, 11.8 HEPES, 10 Glucose. The solution was adjusted to pH 7.4 with NaOH and the final osmolarity was 300–320 mOsm. The washed neurons were incubated with mouse anti-GluA1 (Sigma Cat# ZMS1007; 1:100) in HEPES ACSF supplemented with 2% normal goat serum (NGS) at 37° C for 5 minutes. Coverslips were then gently washed 3x in HEPES ACSF then fixed for 20 minutes in 4% PFA+4% sucrose at room temperature. Coverslips were then washed 3x (10 minutes per wash) with PBS and permeabilized with −20° C methanol for 10 minutes. Permeabilized neurons were blocked (PBS+2% NGS) for 1 hour before incubation with chicken anti-Homer1 (Sysy Cat# 160 00; 1:1000). Neurons were then washed 3x for 10 minutes per wash in PBS, incubated with Aberrior secondary antibodies: goat anti-mouse STAR 635P and goat anti-chicken STAR 580 for 1 hour and washed again in PBS (3x for 10’). Coverslips were mounted on glass slides with Vectashield Hardset mounting media (H-1400–10).

### STED:

STimulated Emission Depletion (STED) imaging was performed on an Abberior Stedycon STED and confocal scanhead system incorporated onto an Olympus IX-83 microscope with an Olympus 100x STED objective (UPLXAPO100XO, NA 1.45, oil). This imaging system has two STED and four confocal imaging channels. Excitation and STED light are generated by the Stedycon pulsed laser system that generates a STED depletion laser at 775nm and excitation lasers at 405nm, 488nm, 561nm and 640nm. Fluorescence bleed-through is mitigated by highly accurate time-gated interleaving of the 561nm and 640nm excitation lasers. Each optical section is subjected to Huygens deconvolution before image analysis.

### STED image analysis:

On average, three randomly selected dendrites (~10 synapses per dendrite) were imaged using STED microscopy. These synapses were subjected to a semi-automated computational analysis pipeline as previously described in detail^116,117^. Briefly, all synapses were manually selected and had to encompass all the GluA1 and Homer1 signal within the Z-stack to be included in the dataset. Nanoscale properties of GluA1 and Homer1 were identified by using an object-based image segmentation approach based on a Split-Bregman image segmentation algorithm, first described in^118^ and subsequently incorporated into the MOSAIC image processing suite for ImageJ/FIJI (http://mosaic.mpi-cbg.de/)^119^. Object boundaries were delineated from background-corrected STED images (using a histogram-based background estimator, also implemented as part of the MOSAIC suite). The parameters used for background subtraction and segmentation were determined experimentally, however, once determined, these parameters were applied to all images and conditions. Binary masks generated in this process were then imported into MATLAB (Mathworks) where the geometric properties of the defined objects were calculated. The GluA1 compartment volume, defined as the synaptic volume occupied by GluA1 at a Homer1 positive synapse, was determined by using the [convhull] function in MATLAB to calculate a convex hull for all GluA1 objects. The nanocluster numbers and nanocluster volumes were determined using the [regionprops] function found in MATLAB’s Image Processing Toolbox. Output metrics were then imported into Prism (GraphPad) for further analysis.

### Primary culture electrophysiology:

Whole-cell voltage-clamp recordings were made from pyramidal neurons, identified by intrinsic membrane properties and mEPSC response kinetics, in all conditions. Recording pipettes were pulled to 3–4 mΩ and series resistance was constantly monitored. Cells that experienced series resistance changes greater than 20% were discarded. Cultures were continuously superfused with room temperature ACSF (in mM): 126 NaCl, 2.5 KCl, 1 NaH_2_PO4, 26.2 NaHCO_3_, 2.5 CaCl_2_, 1.3 MgSO_4_•7H_2_O, 11 D-Glucose, ~290 mOsm. The internal solution consisted of (in mM): 115 Cs-Methanesulfonate, 15 CsCl, 8 NaCl, 0.2 EGTA, 10 HEPES, 4 Mg-ATP, 0.3 Na-GTP, 10 TEA-Cl, 10 Na_2_-phosphocreatine, 1 MgCl_2_, ~290 mOsM_._ Cells were held at −70 mV and mEPSCs (0.5 μM TTX and 100 μM Picrotoxin) were recorded. Miniature events were handpicked and analyzed in Clampfit 10 (Molecular Devices) using template matching and a threshold of 5 pA. For all experiments, the experimenter was blind to the recording condition.

### Expansion microscopy imaging:

Immunostained samples on 12 mm-diameter coverslips were unmounted and washed with PBS for 3 times. 100 mM sodium bicarbonate solution (pH 8.5) was used to pre-incubate the samples for 15 min, followed by incubation with 0.04% (w/v) glycidyl methacrylate (GMA) (Sigma, Cat#151238) in 100 mM sodium bicarbonate for 3 hours at room temperature. After GMA treatment, samples were washed with PBS for 3 times (5 min per wash) and transferred onto a parafilm placed in a dish. The samples were first incubated with monomer solution (8.6 g sodium acrylate, 2.5 g acrylamide, 0.15 g N,N’-methylenebisacrylamide, 11.7 g sodium chloride in 100 ml PBS buffer) on ice for 5 min. Gelation solution (mixture of monomer solution, 10% (w/v) N,N,N′,N′ Tetramethylethylenediamine (TEMED) stock solution, 10% (w/v) ammonium persulfate (APS) stock solution and water at 47:1:1:1 volume ratio) was then quickly added onto the samples (50 μL per sample on coverslip) and incubated on ice for another 5 min. The samples with gelation solution were later transferred to a 37 °C humidity chamber for gelation. After 1 h gelation, the gelated samples were immersed in heat denaturation buffer (200 mM sodium dodecyl sulfate, 200 mM NaCl, and 50 mM Tris pH 6.8) for 1.5 hours at 78°C and washed with excess of DNase/RNase-free water and expanded overnight. Fully expanded gelated samples (~3.6 times expansion) were trimmed and transferred to a poly-lysine-coated glass bottom dish prior to imaging. The imaging of expanded gelated samples was performed on an Airyscan l microscope (ZEISS LSM 980 with Airyscan 2) with a 63x water-immersion objective (Zeiss LD C-Apochromat 63x/1.15 W Corr M27). Airyscan SR and the best signal mode with 0.2 AU pinhole and 1.25 AU total detection area were used for all expansion imaging.

### Behavior:

#### Auditory Fear Conditioning:

Mice were first habituated to the auditory fear conditioning chambers for two days before beginning experiments. Mice were individually placed in the chamber (MedAssociates) located in the center of a sound attenuating cubicle. The conditioning chamber was cleaned with 10% ethanol to provide a background odor. A ventilation fan provided a background noise at ~55 dB. After a 2 min exploration period, three 2 kHz, 85 dB tones were played, for 30s each, with a 90s interval between them. Following these initial tones, three subsequent tones were pained with a 1s, 0.75 mA foot shock. The foot shocks co-terminated with the tone. The mice remained in the chamber for another 60s before being returned to the home cages. The time spend freezing during each of the tones was quantified. For recall testing, the animals were placed into chambers with modified walls, flooring, and scents. After allowing the animals 90 seconds for acclimation to the new chamber, one 30 second tone was presented, and the time spent freezing was quantified.

#### Conditioned Place Preference:

To test for drug-induced CPP, animals were tested in a single drug pairing, two-chamber CPP test. Each chamber was given different wall contexts. On the first day, animals were initially placed into the right chamber, and allowed to freely explore both chambers for thirty minutes (pre-test). On the second day, animals were saline-conditioned to the left side, and on the following day, cocaine-conditioned to the right side. Drug conditioning was counterbalanced across the mice. Intracranial surgeries were then performed on day four. Two days later, animals were again initially placed into the right chamber, and allowed to explore freely (post-test). CPP scores were computed as the subtracted CPP score, which equals time spent in the drug paired chamber [(posttest-pretest)/posttest]. Each session was 30 minutes.

#### T-Maze:

Spatial memory was assessed using the T-maze. Here we assessed learning through the observation that mice performed better on this task when tested daily. Briefly, mice were placed into one arm of a T-maze, where they then entered a second arm. Following this entrance, the next seven entrances into other arms were recorded, with entry into the arm where they had recently come from being recorded as an incorrect spontaneous alternation, and an entry into the arm where they were not was recorded as a correct spontaneous alternation. The number of correct alternations was then divided by seven, and the daily performance for each mouse was reported as a ratio, with 1 being a perfect spontaneous alternation score (7/7 correct). After 3 days of testing, animals were injected with either ZIP, TAT, or vehicle in the dorsal hippocampus. After one additional day of recovery, testing was then performed for three more days.

### Stereotaxic surgeries:

One day following learning, mice were anesthetized using isoflurane anesthesia (4% induction, 1.5% maintenance). Mice were then placed in a stereotaxic apparatus (Stoelting), and injected with a small molecule inhibitor, peptide, or both. Injection coordinates were as follows:

NAcMed: AP +1.55, ML 0.7, DV −4.0;

Amygdala: AP −1.43, ML 2.5, DV −4.5;

Dorsal hippocampus: AP −2.00, ML 1.5, DV −1.5

#### Retroorbital injections:

Mice were briefly anesthetized with 4% isoflurane. While anesthetized, 1 μL of ZIP or TAT peptide was injected retro-orbitally using a Hamilton syringe (Grainger, 49AL65). Mice were then allowed to wake up and resume normal activity.

### Data analyses and statistics:

All statistics were calculated using GraphPad Prism 9 software. Statistical significance between direct comparisons was assessed by unpaired or paired t-tests. When multiple conditions were compared, one- or two-way ANOVAs were first performed, as appropriate, and if significant differences were identified, t-tests were then performed for each individual comparison. Dot plots presented throughout the manuscript include a bar representing the mean value for each group. Error bars represent s.e.m. throughout. For all figures, ns P > 0.05, * P ≤ 0.05, ** P ≤ 0.01, *** P ≤ 0.001, **** P ≤ 0.0001.

## Supplementary Material

Supplement 1
