# Supplementary material for: Cationic peptides erase memories by removing synaptic AMPA receptors through endophilin-mediated endocytosis
Source: Res Sq. 2023 Nov 21:rs.3.rs-3559525. Preprint. [Version 1] doi: 10.21203/rs.3.rs-3559525/v1 (PMC10690331; doi:10.21203/rs.3.rs-3559525/v1)
Supplement: Supplement 1 [file NIHPPrs3559525v1-supplement-1.pdf]

Supplemental Figure Legends:

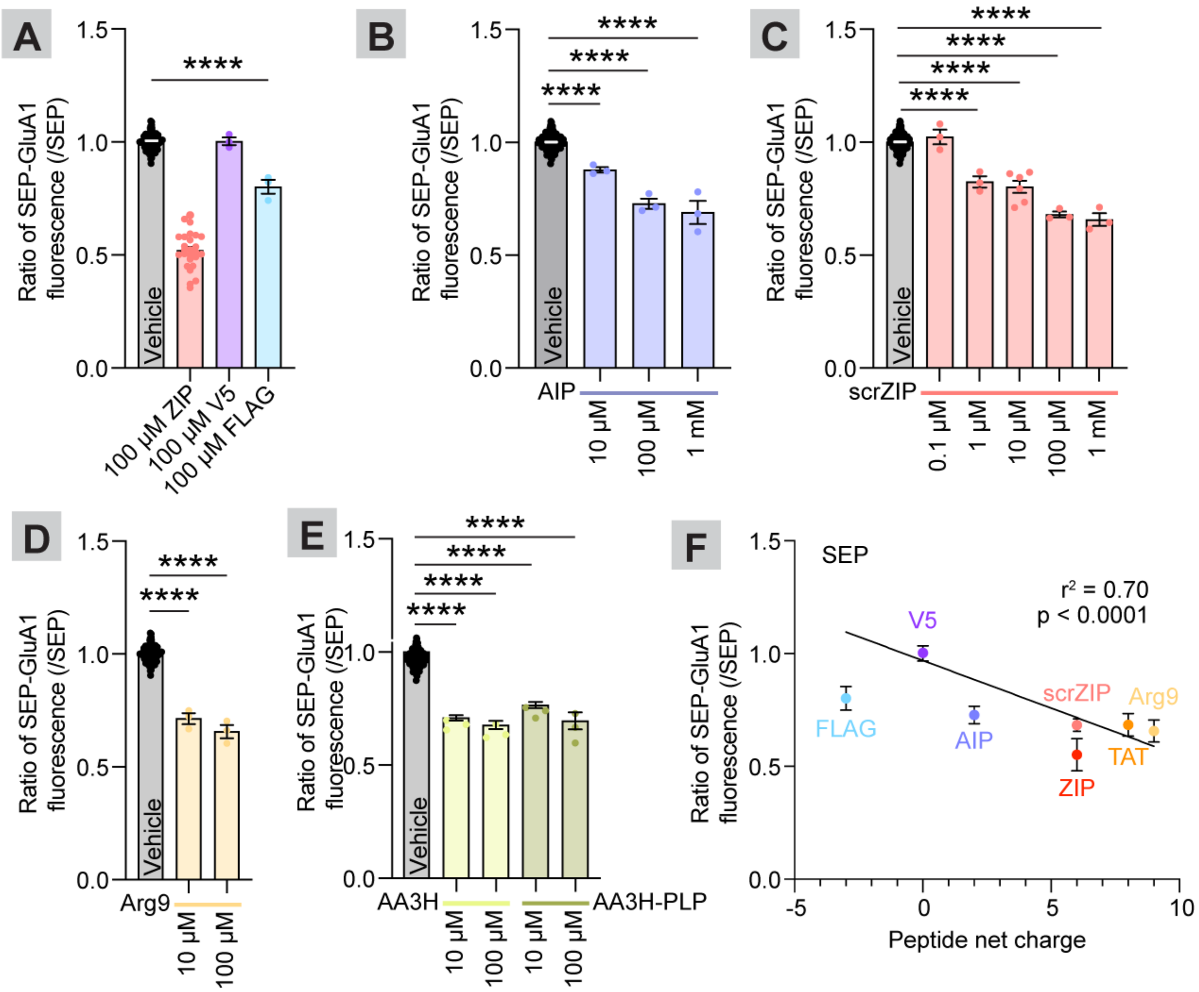

Figure S1: Concentration dependence of various cationic and non-cationic peptides on SEP-GluA1 fluorescence.

(A) Effect of 100 μM V5 or FLAG peptides relative to 100 μM ZIP on SEP-GluA1 fluorescence (Vehicle vs. V5, 1.00 vs. 1.00, p = 0.89, n = 114 and 3, respectively; Vehicle vs. FLAG, 1.00 vs. 0.80, p < 0.0001, n = 114 and 3, respectively).

(B) Concentration dependence of the AIP peptide on SEP-GluA1 fluorescence (Vehicle vs. 10 μM AIP, 1.00 vs. 0.88, p < 0.0001, n = 114 and 3, respectively; Vehicle vs. 100 μM AIP, 1.00 vs. 0.73, p < 0.0001, n = 114 and 3, respectively; Vehicle vs. 1 mM AIP, 1.00 vs. 0.69, p < 0.0001, n = 114 and 3, respectively).

(C) Concentration dependence of the scrZIP peptide on SEP-GluA1 fluorescence (Vehicle vs. 0.1 μM scrZIP, 1.00 vs. 1.02, p = 0.28, n = 114 and 3, respectively; Vehicle vs. 1 μM scrZIP, 1.00 vs. 0.82, p < 0.0001, n = 114 and 3, respectively; Vehicle vs. 10 μM scrZIP, 1.00 vs. 0.80, p < 0.0001, n = 114 and 3, respectively; Vehicle vs. 100 μM scrZIP, 1.00 vs. 0.68, p < 0.0001, n = 114 and 3, respectively; Vehicle vs. 1 mM scrZIP, 1.00 vs. 0.66, p < 0.0001, n = 114 and 3, respectively).

(D) Concentration dependence of the Arg9 peptide on SEP-GluA1 fluorescence (Vehicle vs. 10 μM Arg9, 1.00 vs. 0.71, $p < 0.0001$, n = 114 and 3, respectively; Vehicle vs. 100 μM Arg9, 1.00 vs. 0.66, $p < 0.0001$, n = 114 and 3, respectively).

(E) Concentration dependence of the non-cationic AA3H and AA3H-PLP peptides on SEP-GluA1 fluorescence (Vehicle vs. 10 μM AA3H, 1.00 vs. 0.71, $p < 0.0001$, n = 114 and 3, respectively; Vehicle vs. 100 μM AA3H, 1.00 vs. 0.68, $p < 0.0001$, n = 114 and 3, respectively; Vehicle vs. 10 μM AA3H-PLP, 1.00 vs. 0.77, $p < 0.0001$, n = 114 and 3, respectively; Vehicle vs. 100 μM AA3H-PLP, 1.00 vs. 0.70, $p < 0.0001$, n = 114 and 3, respectively).

(F) Relationship between the net charge of each peptide and the relative change in SEP-GluA1 fluorescence (at 100 μM peptide).

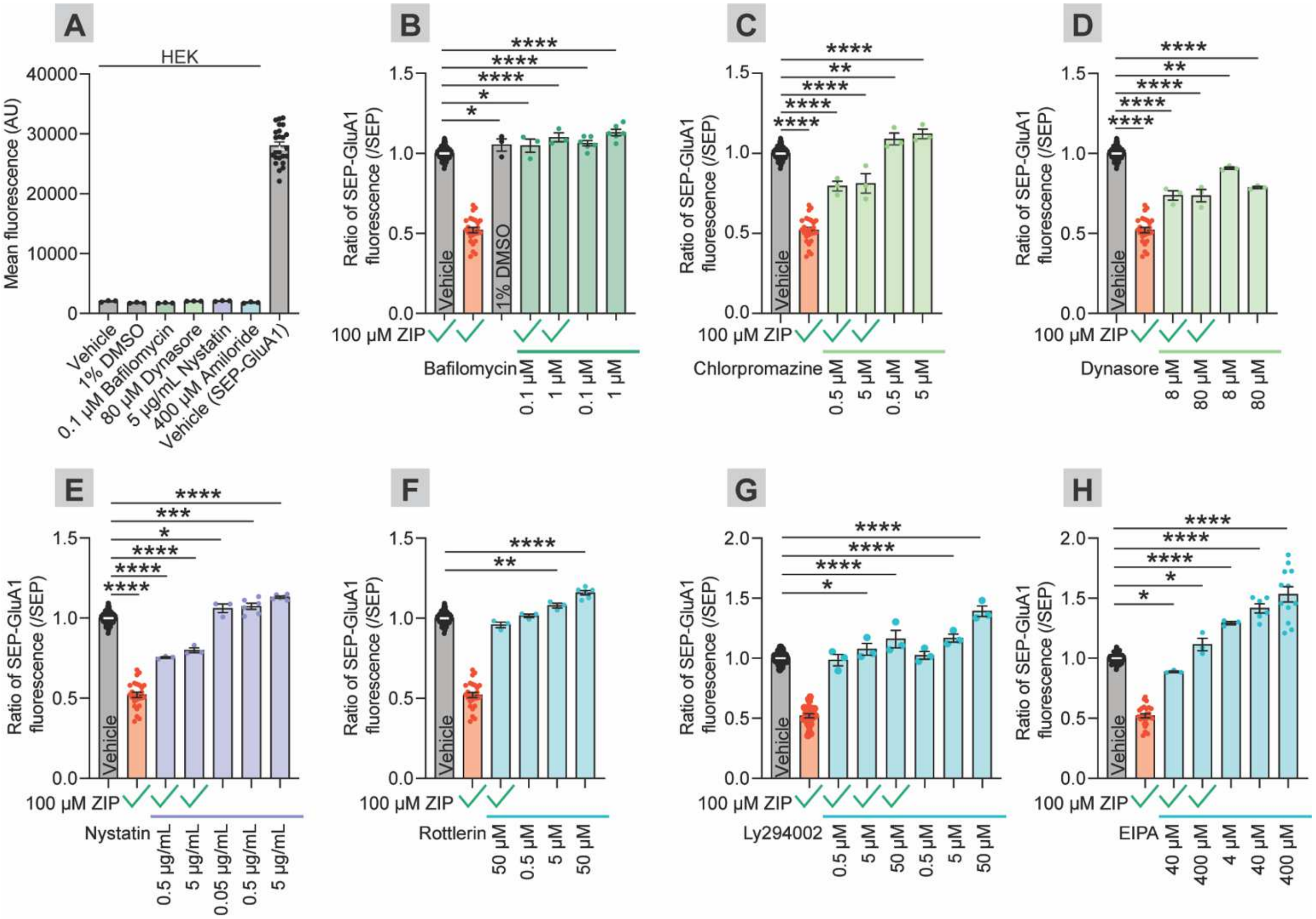

Figure S2: Effects of endocytosis-modulating drugs at different concentrations in HEK-SEP-GluA1 cells.

(A) Effects of various drugs on HEK cells without SEP-GluA1, relative to vehicle-treated HEK-SEP-GluA1 cells. Data are presented as mean fluorescence in the FITC channel per sample.

(B) Effect of bafilomycin at different concentrations, with and without subsequent ZIP administration. As bafilomycin A1 canonically inhibits vacuolar H+-ATPase, which is the main proton pump responsible for endosome acidification, this likely prevent pH-induced changes in GFP fluorescence upon endocytosis (Vehicle (saline) vs. 1% DMSO, 1.00 vs. 1.05, $p = 0.02$, $n = 114$ and 3, respectively; Vehicle (saline) vs. 0.1 μM bafilomycin + ZIP, 1.00 vs. 1.05, $p = 0.03$, $n = 114$ and 3, respectively; Vehicle (saline) vs. 1 μM bafilomycin + ZIP, 1.00 vs. 1.10, $p < 0.0001$, $n = 114$ and 3, respectively; Vehicle (saline) vs. 0.1 μM bafilomycin, 1.00 vs. 1.13, $p < 0.0001$, $n = 114$ and 6, respectively; Vehicle (saline) vs. 1 μM bafilomycin, 1.00 vs. 1.06, $p < 0.0001$, $n = 114$ and 6, respectively). This indicates our assay is working properly.

(C) Effect of chlorpromazine at different concentrations, with and without subsequent ZIP administration (Vehicle vs. 0.5 μM chlorpromazine + ZIP, 1.00 vs. 0.80, $p < 0.0001$, $n = 114$ and 3, respectively; Vehicle vs. 5 μM chlorpromazine + ZIP, 1.00 vs. 0.81, $p < 0.0001$, $n = 114$ and 3, respectively; Vehicle vs. 0.5

μM chlorpromazine, 1.00 vs. 1.09, $p = 0.002$, $n = 114$ and 3, respectively; Vehicle vs. 5 μM chlorpromazine, 1.00 vs. 1.12, $p < 0.0001$, $n = 114$ and 3, respectively).

(D) Effect of Dynasore at different concentrations, with and without subsequent ZIP administration (Vehicle vs. 8 μM Dynasore + ZIP, 1.00 vs. 0.74, $p = 0.03$, $n = 114$ and 3, respectively; Vehicle vs. 80 μM Dynasore + ZIP, 1.00 vs. 0.74, $p < 0.0001$, $n = 114$ and 3, respectively; Vehicle vs. 8 μM Dynasore, 1.00 vs. 0.91, $p = 0.001$, $n = 114$ and 3, respectively; Vehicle vs. 80 μM Dynasore, 1.00 vs. 0.79, $p < 0.0001$, $n = 114$ and 3, respectively).

(E) Effect of nystatin at different concentrations, with and without subsequent ZIP administration (Vehicle vs. 0.5 μg/mL nystatin + ZIP, 1.00 vs. 0.75, $p < 0.0001$, $n = 114$ and 3, respectively; Vehicle vs. 5 μg/mL nystatin + ZIP, 1.00 vs. 0.80, $p < 0.0001$, $n = 114$ and 3, respectively; Vehicle vs. 0.05 μg/mL nystatin, 1.00 vs. 1.06, $p = 0.02$, $n = 114$ and 3, respectively; Vehicle vs. 0.5 μg/mL nystatin, 1.00 vs. 1.07, $p = 0.0002$, $n = 114$ and 6, respectively; Vehicle vs. 5 μg/mL nystatin, 1.00 vs. 1.13, $p < 0.0001$, $n = 114$ and 6, respectively).

(F) Effect of rottlerin at different concentrations, with and without subsequent ZIP administration (Vehicle vs. 50 μM rottlerin + ZIP, 1.00 vs. 0.96, $p = 0.17$, $n = 114$ and 3, respectively; Vehicle vs. 0.5 μM rottlerin + ZIP, 1.00 vs. 1.02, $p = 0.59$, $n = 114$ and 3, respectively; Vehicle vs. 5 μM rottlerin, 1.00 vs. 1.08, $p = 0.008$, $n = 114$ and 3, respectively; Vehicle vs. 50 μM rottlerin, 1.00 vs. 1.16, $p < 0.0001$, $n = 114$ and 6, respectively).

(G) Effect of Ly294002 at different concentrations, with and without subsequent ZIP administration (Vehicle vs. 0.5 μM Ly294002 + ZIP, 1.00 vs. 0.99, $p = 0.61$, $n = 114$ and 3, respectively; Vehicle vs. 5 μM Ly294002 + ZIP, 1.00 vs. 1.08, $p = 0.01$, $n = 114$ and 3, respectively; Vehicle vs. 50 μM Ly294002 + ZIP, 1.00 vs. 1.16, $p < 0.0001$, $n = 114$ and 3, respectively; Vehicle vs. 0.5 μM Ly294002, 1.00 vs. 1.02, $p = 0.42$, $n = 114$ and 3, respectively; Vehicle vs. 5 μM Ly294002, 1.00 vs. 1.17, $p < 0.0001$, $n = 114$ and 3, respectively; Vehicle vs. 50 μM Ly294002, 1.00 vs. 1.39, $p < 0.0001$, $n = 114$ and 3, respectively).

(H) Effect of EIPA at different concentrations, with and without subsequent ZIP administration (Vehicle vs. 40 μM EIPA + ZIP, 1.00 vs. 0.89, $p = 0.02$, $n = 114$ and 3, respectively; Vehicle vs. 400 μM EIPA + ZIP, 1.00 vs. 1.11, $p = 0.01$, $n = 114$ and 3, respectively; Vehicle vs. 4 μM EIPA, 1.00 vs. 1.29, $p < 0.0001$, $n = 114$ and 3, respectively; Vehicle vs. 40 μM EIPA, 1.00 vs. 1.41, $p < 0.0001$, $n = 114$ and 6, respectively; Vehicle vs. 400 μM EIPA, 1.00 vs. 1.53, $p < 0.0001$, $n = 114$ and 12, respectively).

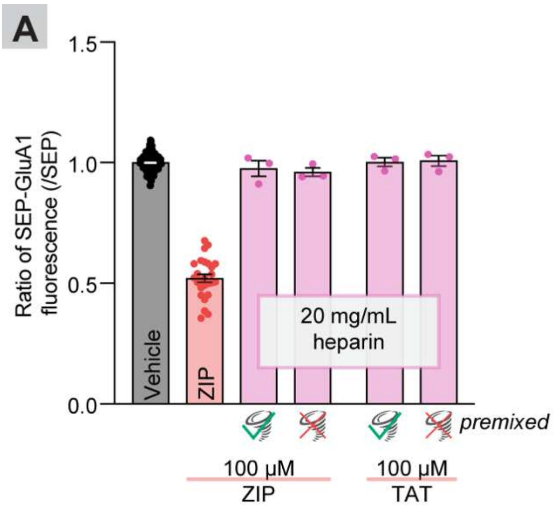

Figure S3: Effect of negatively charged, positively charged, and neutral molecules on SEP-GluA1 fluorescence.

(A) Effect of Heparin on the ZIP-induced reduction in SEP-GluA1 fluorescence, added either to the same tube as ZIP before adding to cells, or added to the cell culture independently before ZIP. Heparin completely blocked ZIP or TAT's effects on SEP-GluA1 fluorescence, whether heparin was premixed with peptide (Vehicle vs. heparin + ZIP, 1.00 vs. 0.98, $p = 0.37$, $n = 114$ and 3, respectively; Vehicle vs. heparin + TAT, 1.00 vs. 1.00, $p = 0.93$, $n = 114$ and 3, respectively) or first added to the cells (Vehicle vs. heparin + ZIP, 1.00 vs. 0.96, $p = 0.15$, $n = 114$ and 3, respectively; Vehicle vs. heparin + TAT, 1.00 vs. 1.01, $p = 0.78$, $n = 114$ and 3, respectively).

(B) Effect of an equivalent concentration of free lysine (100 μM) and arginine (500 μM) as is present in 100 μM ZIP. A minor reduction in SEP-GluA1 fluorescence was observed (Vehicle vs. L-lys/L-arg, 1.00 vs. 0.92, $p < 0.0001$, $n = 114$ and 3, respectively).

(C) Effect of dextrans on SEP-GluA1 fluorescence. No change in SEP-GluA1 fluorescence was observed (Vehicle vs. 0.05 mg/mL, 1.00 vs. 1.00, $p = 0.89$, $n = 114$ and 3, respectively; Vehicle vs. 0.01 mg/mL, 1.00 vs. 0.99, $p = 0.73$, $n = 114$ and 3, respectively; Vehicle vs. 0.5 mg/mL, 1.00 vs. 1.02, $p = 0.37$, $n = 114$ and 3, respectively; Vehicle vs. 1 mg/mL, 1.00 vs. 1.01, $p = 0.54$, $n = 114$ and 3, respectively; Vehicle vs. 5 mg/mL, 1.00 vs. 1.01, $p = 0.65$, $n = 114$ and 3, respectively).

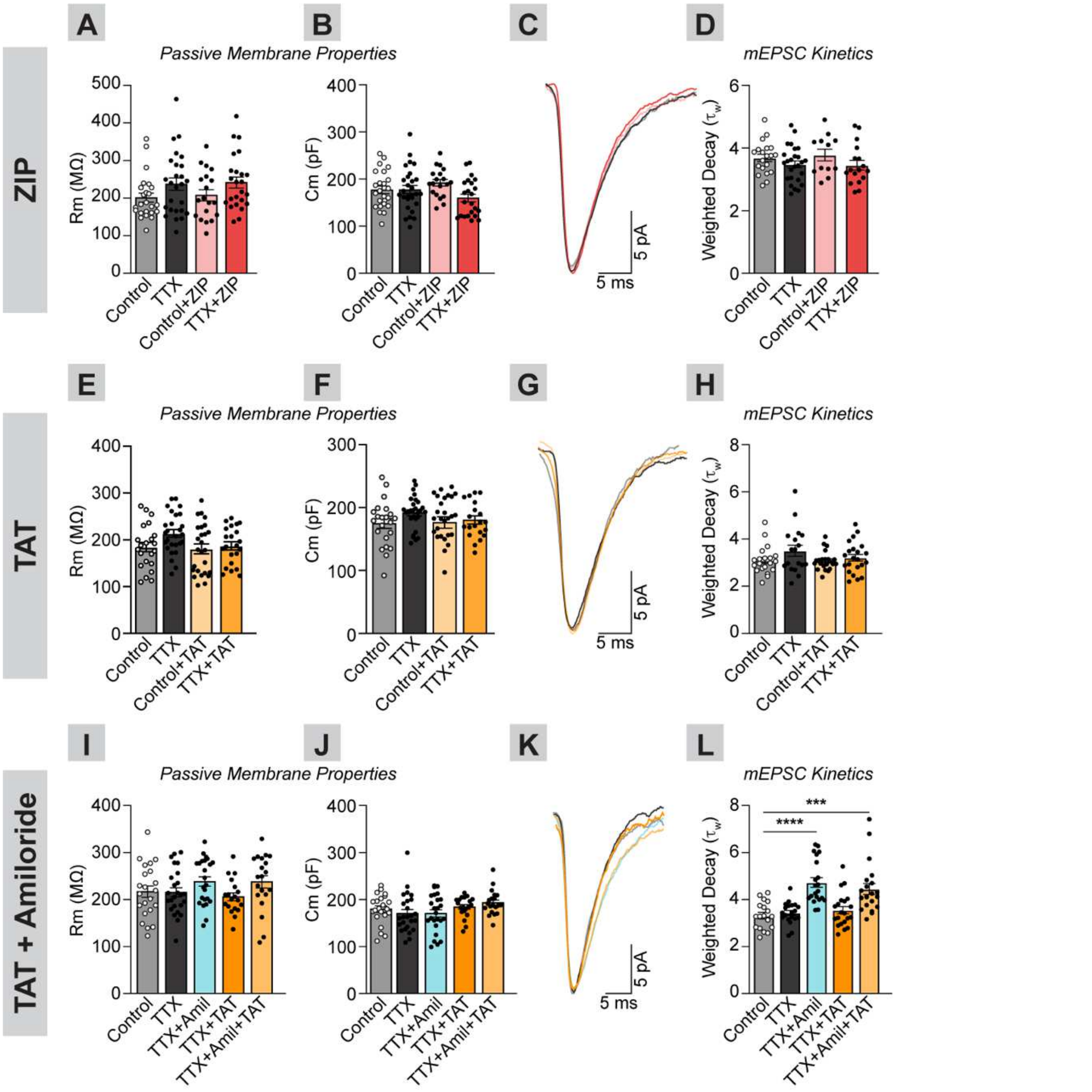

Figure S4: Electrophysiological properties of TTX, ZIP, TAT, and amiloride-treated cultured neurons.

(A) Membrane resistance of ZIP and/or TTX-treated cultures.

(B) Membrane capacitance of ZIP and/or TTX-treated cultures.

(C) Sample EPSCs of each of the ZIP and/or TTX-treated cultures.

(D) Weighted EPSC decay constant for ZIP and/or TTX-treated cultures.

(E) Membrane resistance of TAT and/or TTX-treated cultures.

(F) Membrane capacitance of TAT and/or TTX-treated cultures.

(G) Sample EPSCs of each of the TAT and/or TTX-treated cultures.

(H) Weighted EPSC decay constant for TAT and/or TTX-treated cultures.

(I) Membrane resistance of TAT, amiloride, and/or TTX- treated cultures.

(J) Membrane capacitance of TAT, amiloride, and/or TTX-treated cultures.

(K) Sample EPSCs of each of the TAT, amiloride, and/or TTX- treated cultures.

(L) Weighted EPSC decay constant for TAT, amiloride, and/or TTX-treatment groups. Control vs. TTX, τ_w 3.31 vs. 3.43, $p = 0.66$, $n = 18$ and 20, respectively; Control vs. TTX + amiloride, τ_w 3.31 vs. 4.73, $p < 0.0001$, $n = 18$ and 21, respectively; Control vs. TTX + TAT, τ_w 3.31 vs. 3.55, $p = 0.35$, $n = 18$ and 21, respectively; Control vs. TTX + TAX + amiloride, τ_w 3.31 vs. 4.45, $p < 0.0001$, $n = 18$ and 21, respectively). All other non-noted comparison were non-significant.

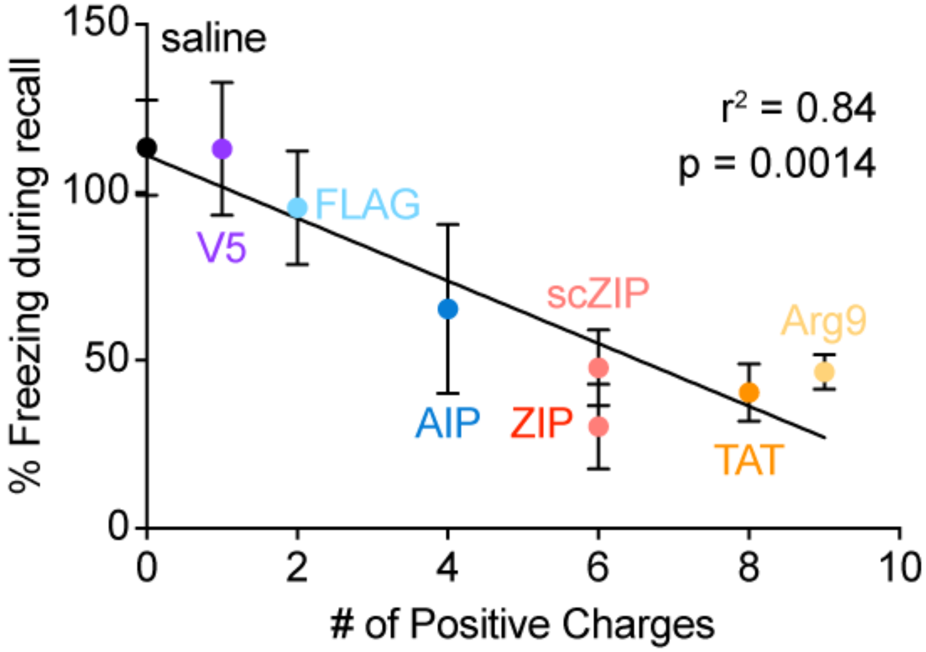

Figure S5: Effect of peptide injection on auditory fear conditioning recall, as a function of the number of cationic charges on the peptide.
